# Dysregulation of protein SUMOylation networks in Huntington’s disease R6/2 mouse striatum

**DOI:** 10.1093/brain/awae319

**Published:** 2024-10-11

**Authors:** Marketta Kachemov, Vineet Vaibhav, Charlene Smith, Niveda Sundararaman, Marie Heath, Devon F Pendlebury, Andrea Matlock, Alice Lau, Eva Morozko, Ryan G Lim, Jack Reidling, Joan S Steffan, Jennifer E Van Eyk, Leslie M Thompson

**Affiliations:** Neurobiology and Behavior, University of California Irvine, Irvine, CA 92697, USA; Advanced Clinical Biosystems Research Institute, The Smidt Heart Institute, Cedars-Sinai Medical Center, Los Angeles, CA 90048, USA; Department of Psychiatry and Human Behavior, University of California Irvine, Irvine, CA 92868, USA; Advanced Clinical Biosystems Research Institute, The Smidt Heart Institute, Cedars-Sinai Medical Center, Los Angeles, CA 90048, USA; Neurobiology and Behavior, University of California Irvine, Irvine, CA 92697, USA; Neurobiology and Behavior, University of California Irvine, Irvine, CA 92697, USA; Advanced Clinical Biosystems Research Institute, The Smidt Heart Institute, Cedars-Sinai Medical Center, Los Angeles, CA 90048, USA; Sue and Bill Gross Stem Cell Center, University of California Irvine, Irvine, CA 92697, USA; Neurobiology and Behavior, University of California Irvine, Irvine, CA 92697, USA; Institute of Memory Impairments and Neurological Disorders, University of California Irvine, Irvine, CA 92697, USA; Institute of Memory Impairments and Neurological Disorders, University of California Irvine, Irvine, CA 92697, USA; Department of Psychiatry and Human Behavior, University of California Irvine, Irvine, CA 92868, USA; Advanced Clinical Biosystems Research Institute, The Smidt Heart Institute, Cedars-Sinai Medical Center, Los Angeles, CA 90048, USA; Neurobiology and Behavior, University of California Irvine, Irvine, CA 92697, USA; Department of Psychiatry and Human Behavior, University of California Irvine, Irvine, CA 92868, USA; Sue and Bill Gross Stem Cell Center, University of California Irvine, Irvine, CA 92697, USA; Institute of Memory Impairments and Neurological Disorders, University of California Irvine, Irvine, CA 92697, USA

**Keywords:** Huntington’s disease, SUMO, proteomics, PIAS1, mGLUR7, E3 SUMO ligase, synaptic

## Abstract

Huntington’s disease is a neurodegenerative disorder caused by an expanded CAG repeat mutation in the Huntingtin (*HTT*) gene. The mutation impacts neuronal protein homeostasis and cortical/striatal circuitry. SUMOylation is a post-translational modification with broad cellular effects including via modification of synaptic proteins.

Here, we used an optimized SUMO protein-enrichment and mass spectrometry method to identify the protein SUMOylation/SUMO interaction proteome in the context of Huntington’s disease using R6/2 transgenic and non-transgenic mice.

Significant changes in the enrichment of SUMOylated and SUMO-interacting proteins were observed, including those involved in presynaptic function, cytomatrix at the active zone, cytoskeleton organization and glutamatergic signalling. Mitochondrial and RNA-binding proteins also showed altered enrichment. Modified SUMO-associated pathways in Huntington’s disease tissue include clathrin-mediated endocytosis signalling, synaptogenesis signalling, synaptic long-term potentiation and SNARE signalling. To evaluate how modulation of SUMOylation might influence functional measures of neuronal activity in Huntington’s disease cells *in vitro*, we used primary neuronal cultures from R6/2 and non-transgenic mice. A receptor internalization assay for the metabotropic glutamate receptor 7 (mGLUR7), a SUMO-enriched protein in the mass spectrometry, showed decreased internalization in R6/2 neurons compared to non-transgenic neurons. SiRNA-mediated knockdown of the E3 SUMO ligase protein inhibitor of activated STAT1 (*Pias1*), which can SUMO modify mGLUR7, reduced this Huntington’s disease phenotype. In addition, microelectrode array analysis of primary neuronal cultures indicated early hyperactivity in Huntington’s disease cells, while later time points demonstrated deficits in several measurements of neuronal activity within cortical neurons. Huntington’s disease phenotypes were rescued at selected time points following knockdown of *Pias1*.

Collectively, our results provide a mouse brain SUMOome resource and show that significant alterations occur within the post-translational landscape of SUMO-protein interactions of synaptic proteins in Huntington’s disease mice, suggesting that targeting of synaptic SUMO networks may provide a proteostatic systems-based therapeutic approach for Huntington’s disease and other neurological disorders.

## Teaser

SUMOylation is a post-translational modification with broad cellular effects. Kachemov *et al.* characterize the protein SUMOylation/SUMO interaction proteome in a Huntington’s disease mouse model, and identify changes in SUMO-associated synaptic proteins that may contribute to disease.

## Introduction

Huntington’s disease (HD) is a devastating neurodegenerative disorder caused by a CAG trinucleotide-repeat expansion mutation in the Huntingtin (*HTT*) gene.^1^ This leads to translation of a mutant Huntingtin (mHTT) protein containing an expanded polyglutamine repeat within the amino terminus. Mutation of the HTT protein leads to a variety of maladaptive protein functions, some of which directly impact brain synaptic function.^2^ For example, mHTT disrupts axonal transport, reducing synaptic vesicle activity and neurotransmitter release.^3,4^ Ultimately, the HD mutation alters neuronal protein homeostasis within the cortico-striatal circuit,^5^ eventually resulting in the loss of medium spiny neurons (MSNs) in the striatum (including the putamen and globus pallidus) and atrophy of the cortex.^6,7^ MSNs constitute 95% of the neuronal population of the striatum in rodents^8^ and receive cortical inputs that release the excitatory neurotransmitter glutamate.^9^ At excessive levels, glutamate can cause neuronal death through glutamatergic excitotoxicity. Experimental studies utilizing glutamate analogues induce a similar pattern of neuronal death observed in HD,^10,11^ suggesting a role for excitotoxicity by glutamatergic cortical inputs in disease pathogenesis. One proposed mechanism through which glutamate excitotoxicity may contribute to HD pathogenesis is the altered metabotropic glutamate receptor signalling seen in various models of HD.^12,13^ The family of metabotropic glutamate receptors (mGLURs) have been linked to excitotoxicity^14^ through their control of membrane enzymes and second messenger systems.^15-18^ Furthermore, regulation of mGLUR localization and function is linked to SUMOylation in neurons.^19^

The small ubiquitin-like modifier (SUMO) protein has emerged as a critical regulator of neuronal development, neuronal stress responses, and synaptic transmission and plasticity.^20^ SUMOylation of target proteins mediates regulation via diverse processes, including synapse formation, mRNA trafficking, neurotransmitter release, synaptic channel activity, receptor endocytosis and synaptic plasticity.^20-25^ SUMOylation occurs when SUMO is covalently conjugated to the side chain of lysine amino acid residues within target substrates through a three-step enzymatic cascade.^26,27^ There are five independent SUMO variants (SUMO1-5),^28^ yet SUMO2 and SUMO3 share 97% sequence homology, are considered functionally equivalent and are referred to collectively as SUMO2/3.^29^ The exposed carboxyl-terminal diglycine motif of a mature SUMO protein, cleaved by a sentrin-specific protease (SENP), is required for activation by the E1 heterodimer enzymes, SAE1 and SAE2. A SUMO protein is then transferred to the sole E2 enzyme, SUMO-conjugating enzyme UBC9 (UBC9), forming a thioester bond between the two molecules. Although UBC9 can independently catalyse SUMO’s conjugation to target substrates, E3 SUMO ligases, such as the Protein Inhibitor of Activated STAT (PIAS) family proteins and RANBP2, facilitate this process and provide target specificity. SUMO conjugation can be reversed by isopeptidases (e.g. SENPs), and free SUMO may be reused for subsequent SUMOylation.^26,27^ The PIAS family, consisting of PIAS1, PIASx (i.e. PIAS2),^30^ PIAS3 and PIASy (i.e. PIAS4), is one group of E3 SUMO ligases that can enhance SUMO modification of cellular protein targets.^31^ PIAS proteins were identified originally for their ability to regulate multiple cellular processes, including transcription, immune responses and cytokine signalling.^30,32^

The impact of SUMO-modification spans across multiple neurodegenerative diseases, where causal disease proteins can be SUMO modified, including huntingtin (HD),^33^ amyloid precursor protein (Alzheimer’s disease^34^), alpha-synuclein (Parkinson’s disease^35^), androgen receptor (SBMA),^36^ ataxin-7 (spinocerebellar ataxia 7, SCA7^37^) and ataxin-1 (SCA-1^38^). A recent study looking at ubiquitous knockout of SUMO1 in the striatum of Q175DN HD-heterozygous mice shows the instrumental effects of SUMO across multiple HD phenotypes, including improvement of age-dependent HD-like motor and neurological impairments, striatal atrophy, inflammatory responses and enhanced autophagic flux.^39^ Our laboratory demonstrated that PIAS1 enhanced HTT modification by both SUMO1 and SUMO2,^40^ and that striatal reduction of PIAS1 was protective in R6/2 HD mice and prevented aspects of pathogenesis, including aberrant mHTT accumulation, pathogenic microglial activation and increased levels of the presynaptic vesicle protein synaptophysin.^41^ PIAS1 knockdown also normalized synaptic transcriptional profiles in the knock-in zQ175 mouse model and human induced pluripotent stem cell (iPSC) models of HD.^42^ However, it is not known how SUMOylation of cellular proteins, or interactions with SUMOylated proteins, is altered in the brain in neurodegenerative diseases such as HD. Given the importance of SUMOylation in neuronal function and synaptic activity, we optimized a discovery-based proteomic profile of SUMO-enriched proteins in male and female brain tissue from rapidly progressing transgenic HD-modelled R6/2 mice.^43^ Outcomes highlighted predominant changes occurring for synaptic proteins within the SUMOome. This work provides a catalogue of SUMO-associated proteins in the mouse striatum and evaluates the impact of the HD mutation within an *in vivo* SUMO network. From these data, we validated selected SUMO-enriched targets and investigated the effect of targeted disruption of the SUMO system on neuronal activities in R6/2 primary neuronal cultures by reducing levels of the E3 SUMO ligase, PIAS1.

## Materials and methods

### R6/2 mice

All experimental procedures were in accordance with the Guide for the Care and Use of Laboratory Animals of the National Institutes of Health, and animal protocols were approved by Institutional Animal Care and Use Committees (IACUC) at the University of California, Irvine (UCI), an AAALAC accredited institution.

For SUMO enrichment, four each of male and female 5-week-old HD-modelled R6/2 or non-transgenic (NT) mice were obtained from the Jackson Laboratory [Cat. No. 006494, B6CBA-Tg(HDexon1)62Gpb/3J, genotype 120 ± 5], housed by sex and genotype and aged to 10 weeks. At age 10 weeks, mice were euthanized by pentobarbital overdose and perfused with 0.01 M PBS. Striatum and cerebral cortex were dissected from each hemisphere and flash frozen. For primary cultures, described later, six 5-week-old female HD ovarian transplant females and six C57BL/6J/BCA male studs were purchased from the Jackson Laboratory, then paired for breeding. R6/2 mice have been described elsewhere in detail.^43^ All mice were housed under a 12-h light/dark cycle with *ad libitum* access to chow and water.

### SUMO enrichment

**Figure 1:**
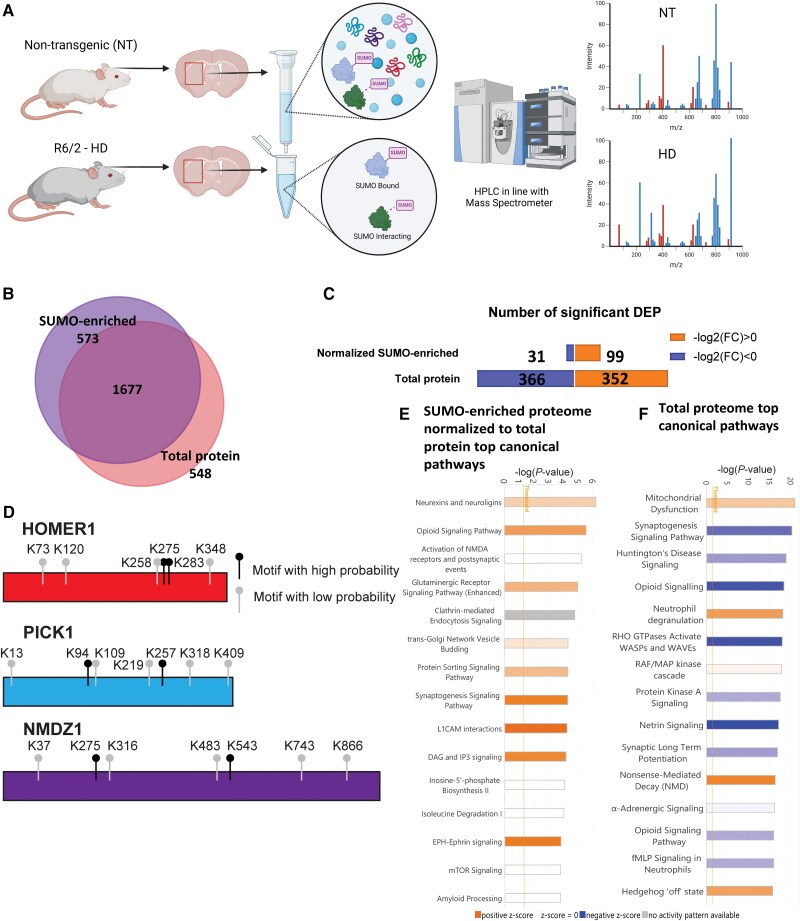
**R6/2 Huntington’s disease mice display altered SUMO-enrichment profiles of synaptic proteins**. (**A**) Schematic of SUMO capture/mass spectrometry approach (*n* = 4/group, males and females). Created in BioRender (BioRender.com/i31q922). (**B**) Overlap between the number of peptides detected from the SUMO-enriched isolation and the peptides from the total striatal lysate. (**C**) Number of unique significantly differentially enriched proteins (DEPs) identified within the SUMO-enriched protein normalised to total protein and the total protein datasets. Values of −log_2_fold-change (FC) > 0 and −log_2_(FC) < 0 indicate higher and lower enrichment in Huntington’s disease (HD) conditions compared to non-transgenic (NT), respectively. (**D**) Representative SUMOylated proteins identified in the discovery-based proteomic experiments and predicted SUMO motifs (high probability in black and low probability in grey) within the HOMER1, PICK1 and NMDZ1 proteins (https://www.abcepta.com/sumoplot). (**E**) SUMO and (**F**) total proteomics of the HD striatum and cortico-striatal connections display significant associations with proteins from synaptic protein pathways. Synaptogenesis signalling was one of the top shared canonical pathways identified between both the normalized SUMO-enriched [−log(*P*-value) = 4.27; positive *z*-score activation of 2.121 SD from the mean] and total protein [−log(*P*-value) = 20.2; negative *z*-score activation of −2.832 SD from the mean] datasets. HPLC = high-performance liquid chromatography.

SUMOylated proteins were enriched from whole striatal tissue in NT and R6/2 mice (males and females, four per group) at 10 weeks of age, a time at which overt symptoms and molecular changes occur, using an in-house optimized enrichment protocol with SUMO Qapture-T kit affinity beads (ENZO Life Sciences, Cat. No. BML-UW1000A). The mouse brain does not lend itself well to subdivision by cyto- or chemoarchitectonic means, and unlike in humans, the caudate nucleus and the putamen are not distinguishable. Therefore, in the rodent striatum, the largest undivided structure is commonly called the caudoputamen (CP).^44^ For SUMO proteomic samples, two striatal hemispheres were homogenized and lysed in 250 μl of lysis buffer (50 mM Tris-HCl, pH 7.5, 150 mM NaCl, 1 × phosphoSTOP pellet/10 ml, 20 mM NaF, 1 μg/ml aprotinin and leupeptin, 0.2 Mm butyric acid, 1 mM PMSF, 25 mM *N*-ethylmaleimide) by miniature tissue douncer (approximately 30 times). Whole-cell lysates were quantified using the Bradford protein assay. For experimental samples, 100 μg total protein lysate was diluted in a final solution of 100 μl volume with binding buffer (50 mM Tris-HCl, pH 7.5), and for positive control samples, 50 μl of purified control SUMO protein was added to 50 μl binding buffer. An aliquot of 30 μg original lysate was retained for whole-cell lysate comparisons. SUMO-Qapture-T SIM-containing affinity matrix (80 μl) was resuspended and washed twice with 200 μl binding buffer. Each sample and control was added to a respective aliquot of washed matrix and rotated at 4°C on a horizontal rotor mixer overnight. Unbound supernatant was removed, and the remaining matrix was washed three times with 200 μl binding buffer. Approximately three-quarters of the SUMO-associated-protein-bound matrix per sample was used for downstream proteomic sample processing (see the ‘SUMO proteomics’ section). The remaining one-quarter of the matrix was boiled at 100°C in a water bath for 10 min with 1× sodium dodecyl-sulfate polyacrylamide gel electrophoresis (SDS-PAGE) gel loading buffer and 1× reducing agent (NuPAGE, Cat. No. NP0009) and SUMO-associated proteins were eluted. See Fig. 1A for the experimental workflow.

### Western blotting

Western blot analysis was used to determine the quantitative levels of SUMO1 (ENZO, Cat. No. BML-PW8330), SUMO2 (ENZO, Cat. No. BML-PW0510A), PIAS1 (Cell Signaling, Cat. No. D33A7 XP), mGLUR7 (EMD Millipore, Cat. No. 07-239) and Myc-tag (Millipore Sigma, Cat. No. 05-419) using specific antibodies according to our previously published protocol and in the Supplementary material.^42^

### SUMO proteomics

The protein-to-trypsin ratio required for sample preparation for mass spectrometry shot-gun proteomics was based on the intensity of total protein quantification following normalization of mass spectra results that demonstrated a median distribution across samples (Supplementary Fig. 1B–D). The SIM-peptide beads bound to SUMO-associated proteins were used for downstream sample processing. SUMO fractions were separated by SDS-PAGE alongside known concentration ranges of whole-cell lysate protein (0.075–3 µg). The amount of starting protein in each fraction was estimated using western blotting-based quantification. SUMO-enriched protein samples were then reduced, alkylated and digested on-bead. Tryptic peptides were resuspended in 0.1% formic acid and analysed on an Ultimate 3000 high-performance liquid chromatography system (Thermo Fisher Scientific) in line with the Orbitrap Fusion Lumos Tribrid Mass Spectrometer (Thermo Fisher Scientific) over a 120-min linear gradient. Mass spectral data were processed in parallel using OMSSA and X!Tandem algorithms against the UniProt database of mouse appended with decoys (Mouse database, March 2016). Search engine results were then converted to pepXML format using omssa2pepXML (v2.1.9) and Tandem2XML (v4.6.0). Peptide spectral match probability scoring was modelled in PeptideProphet (v4.6.0), and the resulting interact.pepXML files of the two search engines were combined in iProphet (v4.6.0). The iprophet pepXML, raw mzML files and mouse FASTA were then imported to Skyline to perform MS1 quantification. Further statistical analysis using MSstats was conducted to determine the relative numbers of unique SUMO-associated proteins and differential enrichment between NT and HD conditions from in-solution digestions of SUMO-enriched samples. Statistical analyses for proteomic results were filtered in MaxQuant using a 0.01 false discovery rate. Protein identifications had a minimum score of 10.5 across all samples analysed. A network-based approach was used to generate a SUMO-associated protein network based on known protein-protein interaction databases (OMICS integrator^45^). Subnetworks and differentially quantified proteins were used as input for GO enrichment analysis (GOrilla), and pathway enrichment analysis using Ingenuity Pathway Analysis (IPA) (Qiagen, v81348237), as well as the protein interaction network extractor (PINE)^46^ for biological interpretation and visualization of the HD SUMOome.

### *In vitro* SUMOylation assays

For *in vitro* SUMOylation assays, HeLa cells were transfected with 2.0 µg of either cDNA construct Myc-PICK1 or Myc-mGLUR7. Three biological replicates for each were also co-transfected with 2.0 µg of his-tagged SUMO1 plasmid or vector control plasmid. A SUMO-limiting condition was created using 2.0 µg of Myc-PIAS1 plasmid, 0.5 µg of His-SUMO1 and 1.5 µg of vector plasmid. Post-transfection, cell media was changed at 24 h, and cells were harvested at 48 h. HeLa cells were lysed under denaturing conditions (lysis buffer: 6 M guanidine HCl, 100 mM NaH_2_PO_4_, pH 7.8, and Tris HCl, pH 7.8). Lysates were sonicated and, for each condition, 2.5% of lysate was subjected to trichloroacetic acid (TCA) precipitation. For His-isolation, 20 μl of magnetic cobalt bead slurry (Dynabeads His-Tag isolation and pulldown, Invitrogen, Cat. No. 10104D) were added to each sample and incubated at room temperature for 1 h. Beads were collected on the magnetic rack, washed twice with Wash Buffer 1 (8 M urea, 100 mM NaH_2_PO_4_, 10 mM Tris-HCl, pH 8), once with Wash Buffer 2 (8 M urea, 100 mM NaH_2_PO_4_, 10 mM Tris-HCl, pH 6.3) and one final time with 1× PBS. Washed beads were eluted in sample loading buffer (10%, Invitrogen, NuPAGE Sample Reducing Agent, 10×, Life Technologies, Cat. No. NP0009), 40% 4× protein loading buffer (LI-COR Biosciences, Cat. No. 928-40004) and 50% MilliQ H_2_O, boiled for 10 min and analysed via SDS-PAGE. Each experiment was performed in triplicate. For mGLUR7 samples, beads were heated at 50°C for 20 min followed by 3 min at 85°C. Experiments were performed at least twice with biological replicates. Qualitative and relative quantitative co-precipitation of target proteins were analysed via western blotting (see earlier). Antibodies for the following antigens were used at the dilutions indicated: Anti-Myc-Tag (Sigma-Aldrich, Cat. No. 05-419-MI, 1:500), Anti-6x-His (Thermo Scientific, Cat. No. PIPA1983B, 1:250), Anti-PIAS1 (Proteintech, Cat. No. 23395-1-AP, 1:1000).

### Primary neuronal cultures

Primary neuronal cultures are described in the Supplementary material, ‘Methods’ section.

### Receptor internalization assay

Receptor internalization assays were performed as described previously and described in the Supplementary material, ‘Methods’ section.^19^ Internalization and surface expression of the receptors were quantified on the branches in a 400 µm^2^ area (of neurites) using the integrated intensities of the labelled signals with Imaris analysis software (Oxford Instruments, v9.9.0). Internalization ratios of Myc-tagged mGLUR7 protein were calculated as the amount of internalized mGLUR7/the amount of total internalized- and surface-expressed receptor.

### Immunofluorescence staining of R6/2 primary neurons

Primary cortical neurons were fixed with 4% paraformaldehyde for 10 min, then washed three times for 5 min with Dulbecco’s PBS (DPBS, Gibco). Cells were incubated in blocking buffer [2% normal donkey serum, 3% bovine serum albumin (Gibco) with 0.05% Tween-20 in DPBS (Sigma)] for 1 h at room temperature. Subsequently, cells were probed overnight at 4°C with the following primary antibodies in blocking buffer: mGLUR7 (Abcam Cat. No. ab233722, 1:200), PSD95 (Abcam, Cat. No. ab12093, 1:250) and β3-tubulin (Synaptic Systems, Cat. No. 302306, 1:500). Cells were then washed three times for 3 min in DPBS and incubated for 90 min in donkey anti-goat 555/chicken 647/mouse 488 IgG 1:500 (Invitrogen, Cat. Nos. A-21432, A78952, A21202) in blocking solution at room temperature. Cells were washed three times for 3 min in DPBS, washed with Hoechst 33258 (1:3000 in DPBS) for 15 min at room temperature and finally washed three times for 3 min in DPBS. Neurons were then mounted with Fluoromount G (Southern Biotech). Maximum projection images were obtained using a 63× 1.3 NA objective on a Zeiss LSM 900 Airyscan 2 confocal microscope.

### Microelectrode array of R6/2 primary neurons

Primary cortical neurons were cultured in 24-well CytoView microelectrode array (MEA) plates (Axion Biosystems, Cat. No. M384-tMEA-24W) at 1 × 10^5^ cells/well (plating only on the electrodes) from postnatal Day 0 (P0) pups as adapted from the manufacturer’s protocol (https://www.axionbiosystems.com/sites/default/files/resources/EX-SeV-CW-Maestro.pdf). MEA measurements were recorded on a Maestro Edge MEA recording system (Axion BioSystems) for 5 min at a time. Recordings were analysed for differences in multiple measures, including spike rate, burst rate and burst frequency using the Axis Navigator software for Maestro MEA systems (v3.6). Baseline MEA measurements of neurons were taken at 5 days *in vitro* (DIV5), followed by treatment with 7.5 µl of 100 µM Accell mouse *Pias1* siRNA SMARTpool (Horizon Discovery, Cat. No. E-059344-00-0020) or Accell non-targeting control pool (Horizon Discovery, Cat. No. D-001910-10-20) in 750 µl 1× siRNA buffer. MEA measurements were recorded every 2–3 days until DIV21, at which point cells were harvested and frozen at −80°C for the protein assay. The MEA metrics that were measured and analysed included: number of spikes; mean firing rate (Hz); weighted mean firing rate (WMFR, Hz); coefficient of variation; number of bursts; burst duration (s); number of spikes/burst; mean inter-spike interval within burst; median inter-spike interval within burst; inter-burst interval (s); burst frequency (Hz); normalized duration interquartile range; inter-burst interval coefficient of variation; burst percentage; and network inter-spike interval coefficient of variation.

### Statistical analyses

Statistical analysis of imaging and microelectrode array experiments was performed with GraphPad Prism 9 (v10.2.2) using a two-way ANOVA with multiple comparisons corrected for with Tukey’s *post hoc* multiple comparisons test. Q-Q plots and Shapiro-Wilk of residuals were used to determine normality. Degrees of freedom and *F*-statistics are reported in the figure legends.

## Results

### R6/2 Huntington’s disease mouse striatum displays altered SUMO-enrichment profiles of synaptic proteins

To investigate alterations in SUMO networks *in vivo* in an HD animal model, we used R6/2 transgenic mice, which express exon 1 of the human *HTT* gene containing approximately 120 ± 5 CAG repeats, providing a robust and accelerated model for assessing HD-associated phenotypes *in vivo*.^43,47^ We used this HD model given its similarities to symptomatic human HD, with significant changes in striatal gene and protein expression consistent with human post-mortem HD tissue^48^ and our previous work showing that modulation of the E3 SUMO ligase, PIAS1, has a significant impact on R6/2 phenotypes.^41^ Striatal tissue was analysed to provide a proteomic snapshot of the SUMOome in the tissues most overtly affected in HD, which included the cortico-striatal synapses.^49^ A method to enrich SUMOylated and SUMO-associated proteins from brain tissue was optimized in conjunction with downstream detection by western blot and mass spectrometry (Supplementary Fig. 1A–D). Striatal dissections were performed on male and female NT and R6/2 mice (*n* = 4/group) as described^42^ at 10 weeks of age, when mice are symptomatic and display transcriptional dysregulation.^41,43,48^ SUMOylated and SUMO-associated proteins were enriched using the SUMO Qapture-T kit, which uses SUMO-interacting motifs (SIMs), consisting of a four-residue-long hydrophobic stretch of amino acids that bind SUMO proteins.^50^ An Orbitrap Fusion Lumos Tribrid Mass Spectrometer was used to acquire data that were then processed as described in the ‘Materials and methods’ section. SUMO1 and SUMO2/3 peptides were successfully detected in proteomic results from the striatal mouse tissue, supporting capture and spectrum matches (Supplementary Fig. 1E). A schematic overview of the proteomic experimental workflow is depicted in Fig. 1A.

Across the combination of male and female samples, we identified 2225 unique protein IDs in the total protein dataset and 2250 in the SUMO-enriched dataset, with an overlap of 1677 (Supplementary material, Files 010 and 011 and Fig. 1B). Post-normalization of the SUMO-enriched proteins to the total protein dataset, 130 unique proteins were significantly differentially enriched (*P* < 0.05) between HD and NT mice (Supplementary material, File 012), and 718 unique proteins were significantly differentially expressed (*P* < 0.05) in the total protein dataset (Supplementary material, File 013), as measured by log_2_ fold-change (log_2_FC). In the total protein dataset, proteins were evenly distributed between up- and down-enrichment, but there were 3-fold more upregulated than downregulated differentially normalized SUMO-enriched proteins (Fig. 1C). We found significant changes in known SUMOylated proteins involved in synaptic function and glutamatergic signalling, such as syntaxin1A,^51^ mGLUR7^19^ and synapsin-1a.^52^ At the time of our analysis, HOMER1 and GLUN1 had not been identified as SUMO targets, but these were altered and contained multiple high-probability SUMOylation motifs (SUMOplot Analysis Program, https://www.abcepta.com/sumoplot) (Fig. 1D). HOMER1 and GLUN1 have now been shown to be SUMO modified,^53^ providing additional validation for our approach. We also identified previously unreported SUMO-associated proteins, including protein interacting with C kinase (PICK1), which has two high probability SUMOylation motifs (Fig. 1D).

**Figure 2:**
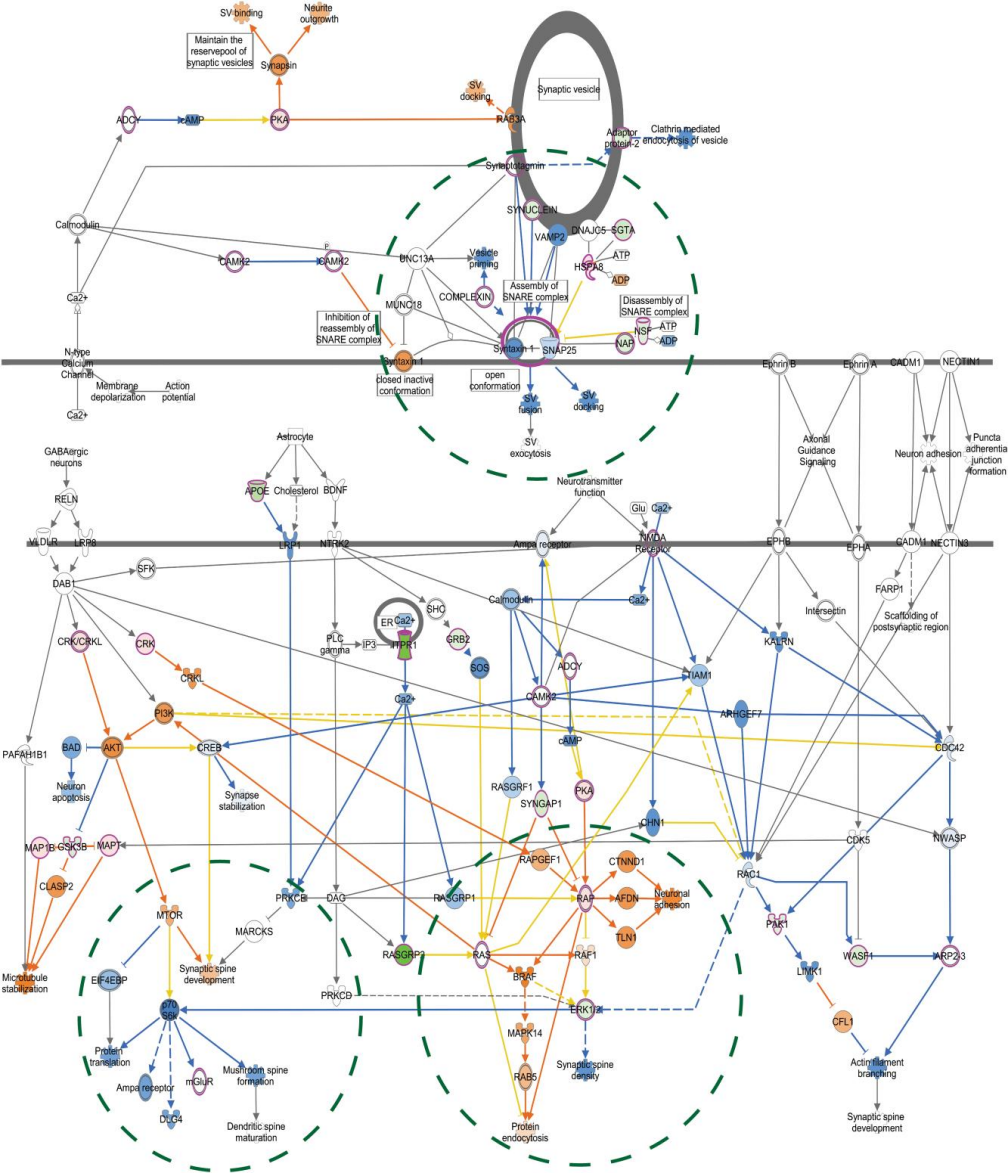
**Ingenuity pathway analysis of differentially enriched proteins.** Significantly differentially enriched proteins from the normalized SUMO-enriched dataset overlaid on the synaptogenesis signalling pathway include CACNA2D2, EPHA4, GRIN1 (NMDZ1), GRM5, NAPG, PRKAR1A, PRKCE, RAC1 and SYT7 (outlined in pink), although other proteins were observed. Darker orange colours indicate greater predicted activation states, while darker blue colours indicate greater predicted inhibition.

The normalized SUMO-enriched and total protein datasets shared some of the same pathways, with synaptogenesis signalling among the highest-ranking canonical pathways [normalized SUMO-enriched protein: −log(*P*-value) = 4.27; positive *z*-score activation of 2.121 SD from the mean. Total protein: −log(*P*-value) = 20.2; negative *z*-score activation of −2.832 SD from the mean] (Fig. 1E and F). IPA also revealed several other significant synaptic-related pathways, including clathrin-mediated endocytosis signalling and synaptic long-term potentiation. The following nine peptides from the SUMO-enriched dataset mapped to the synaptogenesis signalling pathway (Fig. 2) (gene names for the corresponding proteins are listed in parentheses): voltage-dependent calcium channel subunit alpha-2/delta-2 (*Cacna2d1*); EPHA4 (*Epha4*); NMDA receptor NMDZ1 (*Grin1)*; type 5 metabotropic glutamate receptor mGLUR5 (*Grm5*); gamma-soluble NSF attachment protein SNAP-gamma (*Napg*); cyclic AMP-dependent protein kinase type I-alpha regulatory subunit (*Prkar1A*); nPKC-epsilon (*Prkce; Kpce*); Rho family of GTPases Rac1 (*Rac1*); and synaptotagmin-7 (*Syt7*). The relationships between these proteins and others are predicted based on IPA’s advanced protein network modelling. Three subnetworks were identified with predicted association of: (i) several synaptic vesicle docking proteins, i.e. the syntaxin1a (*Stx1A*), synaptosome associated protein 25 (*Snap25*), vesicle associated membrane protein 2 (*Vamp2)* complex; (ii) key molecules for glutamatergic transmission, i.e. AMPA receptors, mGLURs and the NMDAR-postsynaptic-scaffolding-protein, PSD-95 (*Psd95*); and (iii) MAP kinase signal transduction pathway-related proteins, i.e. ERK1/2, MAPK14 and receptor-associated protein (RAP). Previously, SUMOylation has been implicated in all three processes.^19,51,54,55^ For example, SUMOylation of syntaxin1a regulates presynaptic endocytosis,^51^ SUMOylation of mGLUR7 (GRM7) regulates receptor localization on the synaptic membrane^19^ and activation of the ERK pathway causes de-SUMOylation of key transcription factors such as ELK-1.^56^ Additionally, we observed mitochondrial dysfunction in the HD total SUMO proteome (Supplementary Fig. 2).

**Figure 3:**
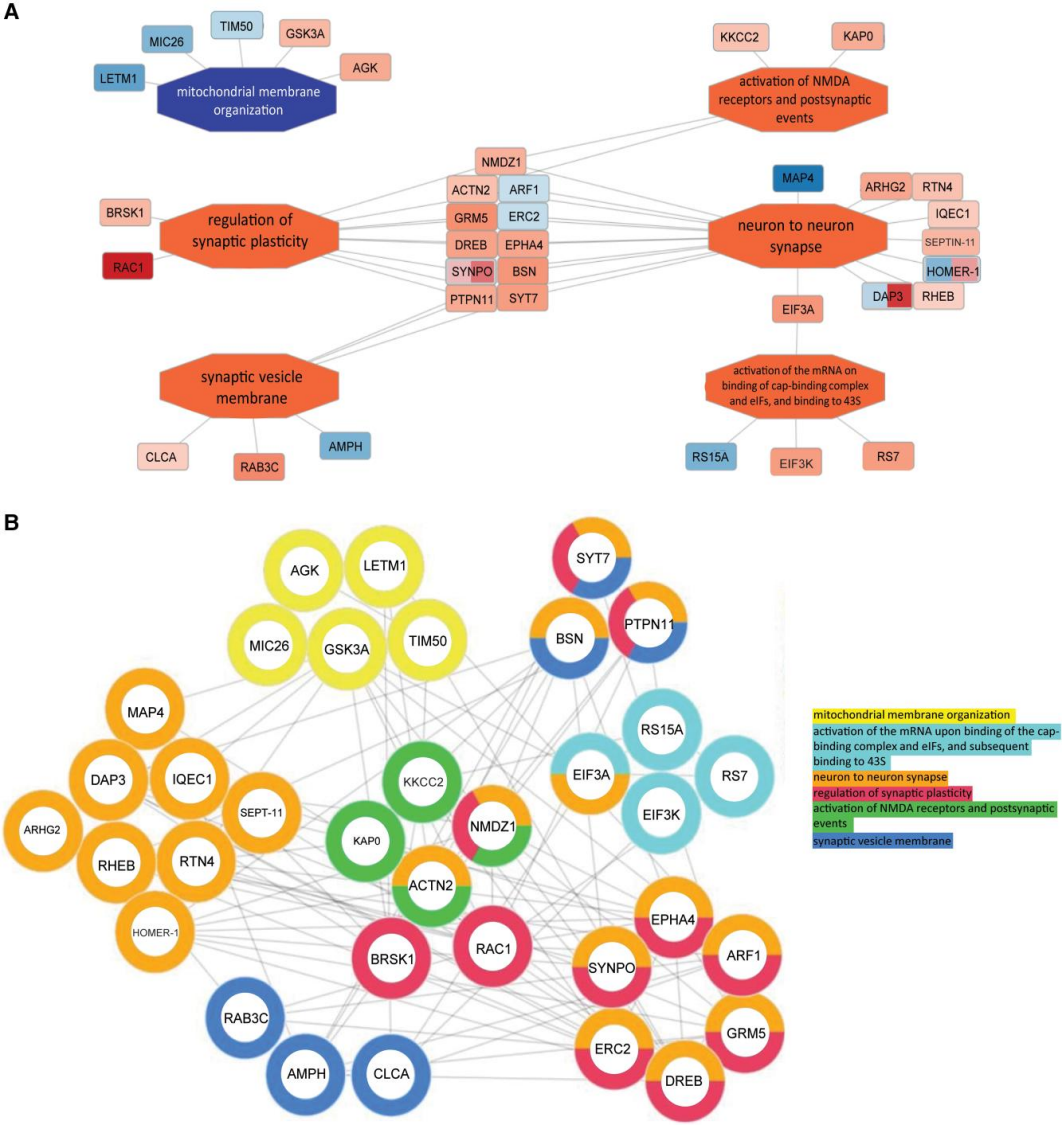
**Visualization analysis of pathways identified from normalized, significantly differentially-SUMO-enriched proteins**. (**A**) An ontology network, constructed from the protein interaction network extractor (PINE) tool, yielded enrichment-related relationships based on peptide-level fold-changes for Huntington’s disease (HD) versus non-transgenic (NT) conditions between the following Gene Ontology terms: regulation of synaptic plasticity; synaptic vesicle membrane; activation of NMDA receptors and postsynaptic events; neuron to neuron synapse; and activation of the mRNA on binding of cap-binding complex and eIFs and binding to 43S pathways. The fold-change of each peptide is projected onto its corresponding protein and is represented in red (for upregulated peptides) and/or blue (for downregulated peptides); the darker the shade, the greater the degree of fold-change between the HD versus NT conditions. For proteins with more than one significantly differentially expressed peptide (for example, SYNPO), the node is sectioned into multiple peptides, with each section depicting the fold-change associated with its corresponding peptide. Additionally, the central enrichment nodes (showing the various ontologies) are in orange or blue to depict overall up- or down-regulation, respectively, based on whether the majority of their associated peptides were up- or down-regulated. (**B**) The doughnut network depicts protein–protein interactions for proteins associated with the pathway terms in **A**. Each protein is shaped in the form of a doughnut, with the colour of the doughnut representing the associated pathway terms. For proteins associated with more than one pathway term (for example, ACTN2), the doughnut is sectioned into multiple colours, each representing a different pathway term.

We next analysed and visualized synaptic pathways in the normalized SUMO-enriched dataset using the PINE tool.^46^ There were relationships between additional synaptic pathways further corroborating the results, and included terms such as regulation of synaptic plasticity, synaptic vesicle membrane, activation of NMDA receptors and postsynaptic events, and neuron to neuron synapse (Fig. 3A). These four pathways highlighted the overlap of several key synaptic proteins responsible for presynaptic function and cytomatrix at the active zone (ERC2, BSN, SYT7), cytoskeleton organization [ACTN2, DREB (*Dbn1*), SYNPO] and glutamatergic transmission [ARF1, NMDZ1 (*Grin1*), GRM5 (mGLUR5, *Grm5*)]. All of these proteins displayed an increased SUMO-enrichment in HD, with the exception of ARF1 and ERC2. The relationships between these molecules and their shared pathways were also mapped within a split-doughnut network (Fig. 3B). These proteins, identified as significantly differentially enriched in the normalized comparison between NT and HD, were found to have multiple connections to previously identified targets of SUMOylation and to the predicted associated network of proteins identified by IPA in Fig. 2.^51,57-67^ As discussed, significant evidence from proteomic analysis indicates SUMO’s role in synaptic protein networks, but the significantly differentially-enriched SUMO proteomic dataset also highlighted proteins of other biologically-relevant themes to HD, including mitochondrial proteins (18/130, 14%), RNA-binding proteins (9/130, 7%) and calcium-related proteins (11/130, 8%) (Supplementary material, File 012). Together, these proteomic analyses indicate that substantial alterations occur within the post-translational modification landscape and SUMO protein interactions for synaptic proteins in HD.

### Comparison of the non-transgenic and R6/2 SUMO datasets to previously published SUMO proteomes

SUMO proteome analysis has not previously been reported for HD brain tissue; however, the SUMO-associated proteins we identified in brain tissue are supported in the SUMO proteomic literature. Our complete list of SUMO-enriched proteins had a 33% (264/792) overlap of SUMOylated proteins from a study conducted by Pronot *et al*.,^53^ which investigated the SUMO profile of synaptosomal proteins from rat forebrain tissue. This study utilized a denaturing immunoprecipitation technique that likely selected for covalent interactions, while eliminating non-covalent SUMO protein interactions. The present study surveyed both covalent and non-covalent SUMO protein interactions, suggesting that at least one-third of the current SUMO-associated proteins identified here are in a covalent interaction with the SUMO protein, and thus are a result of direct SUMO-modification. Studies by Yang *et al*.^68^ and Tirard *et al*.^69^ in transgenic mouse cortical tissue utilized tagged variants of SUMO, allowing the capture of both covalent and non-covalent interactions, similar to the present study. Even greater overlaps were seen for mice from the study by Yang *et al*.^68^ (79%, 89/112) as well as P10 (39%, 114/291) and adult (58%, 81/139) mice analysed by Tirard *et al*.^69^ A SUMO proteomic study in HEK293 cells by Tammsalu *et al*.^70^ identifying proteome-wide SUMO2 modification sites indicated an overlap with the SUMO-enriched dataset presented here [79 of 529 proteins, 52 (66%) of which were associated with RNA-binding molecular function]. Finally, a SUMO proteomic study in HeLa cells by Gonzalez-Prieto *et al.*^71^ identifying non-covalent SUMO interaction networks with DNA damage proteins showed an overlap of all 379 proteins identified in their study with our SUMO-enriched dataset, collectively demonstrating that our method for SUMO enrichment and detection is supported across tissue types (Supplementary material, Files 014 and 015).

### mGLUR7 and PICK1 SUMOylation validation in HeLa cells

**Figure 4:**
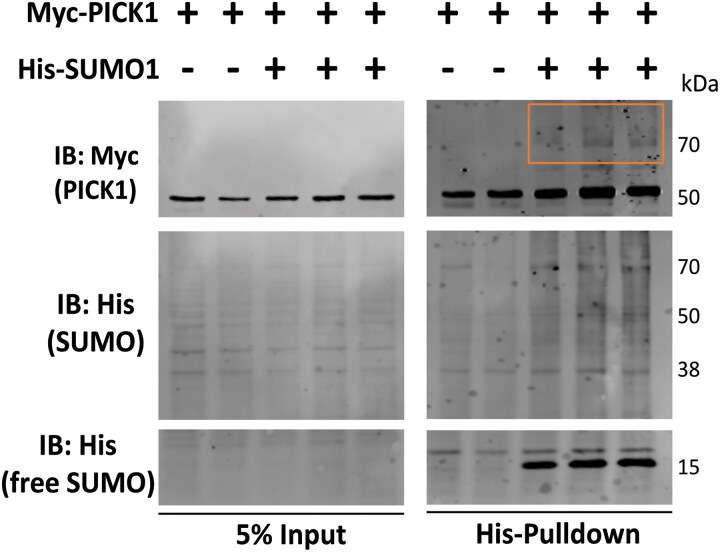
**Validation of PICK1 SUMOylation.** In-cell HeLa SUMOylation assay showing a Myc-tagged PICK1 protein is SUMO modified by His-tagged SUMO1. SUMOylated proteins were enriched via a denaturing His-tag isolation. The area outlined in orange indicates SUMOylated PICK1, which has a higher molecular weight than negative His-SUMO1 controls. The full blot is provided in Supplementary Fig. 3. *n* = 2 for Myc-PICK1-only transfection, *n* = 3 for Myc-PICK1 and His-SUMO1 transfections.

**Figure 5:**
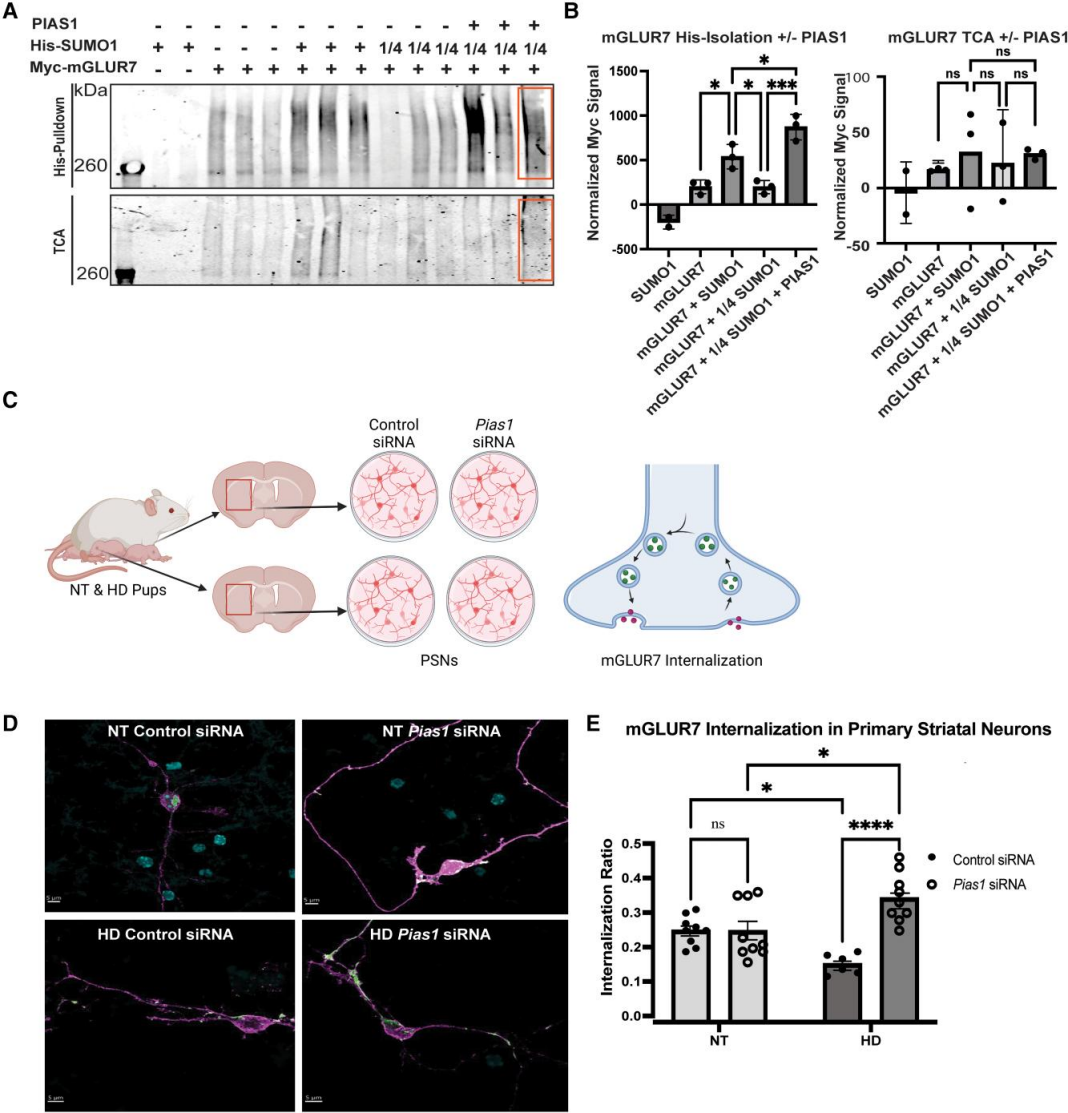
**mGLUR7 SUMOylation and endocytosis**. (**A**) SUMO-limiting experiment in HeLa cells transfected with His-SUMO1, Myc- mGLUR7 and PIAS1. *Top*: His-pulldown of His-SUMO1 protein. *Bottom*: Trichloroacetic acid protein precipitation (TCA) whole-cell lysate. Full SUMO transfections (e.g. ‘+’) included 2 µg of SUMO1 per replicate, whereas one-quarter SUMO1 (e.g. ‘1/4’) were transfected with 0.5 µg of SUMO1 plasmid. The area outlined in orange is an example of the area used to quantify each lane. The full blot is provided in Supplementary Fig. 3. (**B**) Quantification of the Myc-mGLUR7 signal is shown in the His-isolated (*left*) and whole-cell lysate (*right*) conditions. His-isolated samples showed a significant difference in levels of SUMO-mGLUR7 isolated. *F*(4,9) = 34.42, *P* < 0.0001 with Šídák’s multiple comparisons test: mGLUR7 versus mGLUR7 + SUMO1, *P*_adj_ = 0.0173; mGLUR7 + SUMO1 versus mGLUR7 + 1/4 SUMO1, *P*_adj_ = 0.0165; mGLUR7 + 1/4 SUMO1 versus mGLUR7 + 1/4 SUMO1 + PIAS1, *P*_adj_ = 0.0001; mGLUR7 + SUMO1 versus mGLUR7 + 1/4 SUMO1 + PIAS1, *P*_adj_ = 0.0190; analysed by one-way ANOVA. TCA samples show no change in mGLUR7 levels. One-way ANOVA with Šídák’s multiple comparisons test: *F*(4,9) = 0.6085, *P* = 0.6668. *n* = 2 for His-SUMO1-only control transfections, *n* = 3 transfections for all other conditions. (**C**) Schematic of mGLUR7 internalization experiment. Created in BioRender (BioRender.com/i50e678). (**D**) The endocytosis of mGLUR7 was assessed by antibody uptake internalization assay. Non-transgenic (NT) and Huntington’s disease (HD) primary striatal neurons (PSNs) from R6/2 pups were treated with *Pias1* or control siRNA, transfected with Myc-tagged mGLUR7, labelled with anti-c-Myc antibody, washed and returned to conditioned media at 37°C for 15 min. Representative images are of transfected mGLUR7 receptor internalization. Internalized receptors, green; surface receptors, magenta (false-coloured from red for clarity); nuclei, blue. The internalization ratio was the fluorescence intensity of the internalized receptor (green) over the total amount of receptor (sum of green and red intensity shown in magenta). Individual channels are shown in Supplementary Fig. 6. (**E**) Receptor internalization assay for mGLUR7 in R6/2 primary striatal neurons (PSNs) demonstrated a significant decrease in mGLUR7 internalization in HD neurons compared to NT cells. Treatment by *Pias1* siRNA-mediated knockdown significantly reversed levels of mGLUR7 internalization. mGLUR7 internalization: treatment, *F*(1,29) = 18.81, *P* = 0.0002; genotype, *F*(1,29) = 0.001509, *P* = 0.9693; analysed by two-way ANOVA. *P < 0.05, ***P < 0.001, ****P < 0.0001, *n* = branch areas, three per neuron. Graphs represent mean ± standard error of the mean.

SUMO proteomic pathways differentially enriched in HD included the proteins ARF1, PICK1, NMDZ1 and GRM5, all of which take part in glutamatergic signalling, either via AMPA receptor trafficking or direct activation by glutamate.^72-74^ Multiple mGLUR subtypes were identified within the SUMO proteomic dataset, including mGLUR1, 2, 3, 5 and 7. The mGLUR7 protein, a presynaptic receptor for glutamatergic signalling,^75^ has previously been identified as a direct target of SUMOylation, and SUMOylation of mGLUR7 is critical for its localization either on the membrane or internally in the synapse.^76^ Localization of *mGluR7* mRNA has also been shown in the rat cortico-striatal pathway as well as striatopallidal and striatonigral projections.^77^ Inhibiting SUMOylation of mGLUR7 directly increases its internalization, potentially modulating the availability of receptors for glutamatergic neurotransmission. PICK1 also binds to the C terminus of mGLUR7, where it regulates PKCα-evoked phosphorylation of mGLUR7,^78^ and has been demonstrated to function as an endocytic accessory protein required for activity-dependent AMPAR endocytosis.^79^ Using a HeLa cell-based *in vitro* SUMOylation assay,^40,42^ Myc-PICK1 or Myc-mGLUR7 were expressed in HeLa cells alone or with His-SUMO1. Lysates were processed under denaturing conditions and enriched for His-tagged proteins. We detected a novel target of SUMOylation and PICK1 (Fig. 4) and verified the previously identified SUMOylation of mGLUR7 (Fig. 5A and B), i.e. a mobility shift was seen for both Myc-PICK1 and Myc-mGLUR7 in the presence of SUMO1 by western analysis.

Given data showing that mGLUR7 can interact with PIAS1^80^ and our evidence that PIAS1 is a modulator of HD phenotypes,^41,42^ we also evaluated whether PIAS1 could enhance SUMO1 modification of mGLUR7 in the HeLa cell SUMOylation assay. Myc-mGLUR7 and limiting amounts of His-SUMO1 as described^40^ were used to enable detection of increased SUMOylation in the presence of PIAS1. PIAS1 overexpression led to an increase in intensity of higher molecular weight mGLUR7 species (Fig. 5A and B), suggesting an enhancement of mGLUR7 SUMO modification by PIAS1.

### mGLUR7 shows altered internalization in HD primary neurons that is rescued by PIAS1 knockdown

We next sought to evaluate whether a known target of SUMOylation from our SUMO proteomics dataset could inform SUMO-associated changes in proteins implicated in neuronal activity, observed through the lens of glutamatergic signalling and perturbation of the relevant E3 SUMO ligase. We adapted a receptor internalization assay protocol^19^ (see Fig. 5C for experimental workflow) to assess the effect of siRNA mediated *Pias1* knockdown (shown in Supplementary Fig. 4) on mGLUR7 internalization within R6/2 and NT primary striatal neurons (PSNs). PSNs were cultured and treated with either control or *Pias1* siRNA, followed by transfection with a Myc-mGLUR7 construct. Myc-mGLUR7 protein was identified using a Myc primary antibody and external surface receptors stained with a red-fluorescence secondary antibody. Cells were permeabilized, and internalized receptors were then differentiated with a green-fluorescence secondary antibody. Representative images of individual neurons selected for analysis can be seen in Fig. 5D. The ratio of receptor internalization to surface expression was calculated as the expression of internalized receptor signal (green) divided by the total internal and surface expression. HD cells displayed a significantly reduced amount of mGLUR7 internalization compared to NT cells (Fig. 5E). Our findings in HD primary neuronal cultures coincide with older studies implicating the potential importance of mGLUR7 in HD-related pathways. For example, mGLUR7 localizes in presynaptic and postsynaptic terminals of the striatum, globus pallidus and substantia nigra pars reticulata, suggesting its crucial role in modulating neurotransmitter release in major basal ganglia pathways.^77^ Reductions in internalization of mGLUR7, an autoreceptor, may reflect attempts of the neurochemically dysfunctional HD circuit to decrease overactive glutamatergic signalling in these regions by inhibiting further neurotransmitter release.^75,81^ Treatment with *Pias1* siRNA significantly prevented the decreased receptor internalization observed in HD (Fig. 5E). These results indicate that perturbation of the neuronal SUMO-protein-interacting network via an E3 SUMO ligase may provide a viable means for modulating mGLUR7-mediated glutamatergic signalling.

### Neuronal activity is altered in HD primary cortical neurons and partially rescued by PIAS1 knockdown

Glutamatergic signalling and neurotransmission were highly enriched processes within the SUMO proteomics dataset. Activation of mGLUR5, which was differentially SUMO enriched in the dataset (Supplementary Table 3), promotes transient synaptic trapping of UBC9 (the sole SUMO-conjugating enzyme) in dendritic spines, resulting in activity-dependent modulation of global synaptic protein SUMOylation,^82^ suggesting that SUMOylation may drive a mechanism for widespread adaptive tuning of electrical excitability of cells. We predicted that inhibiting SUMOylation of synaptic proteins involved in glutamatergic signalling could correspond to electrophysiological changes in neuronal activity in HD. Significant changes in network-level activity have been well-documented in the cortex^83^ and striatum^84^ of various HD mouse models, such as R6/2, YAC128 and CAG140 knock-in mice, potentially due to alterations in glutamatergic inputs from the cortex into the striatum.^85-88^

**Figure 6:**
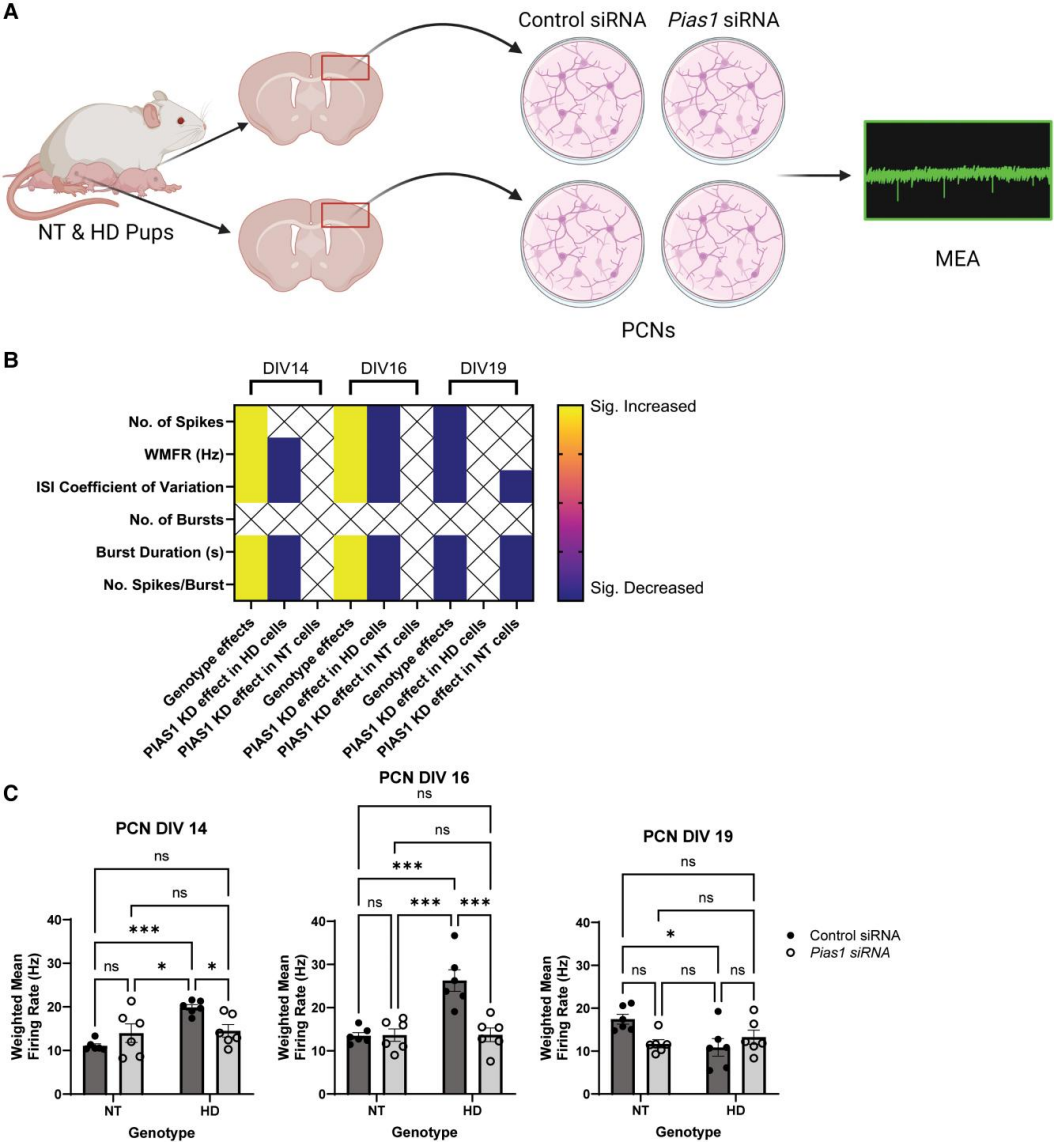
**PIAS1 knockdown rescues alterations to neuronal activity in Huntington’s disease primary cortical neurons**. (**A**) Experimental schematic of microelectrode array (MEA) workflow. Created in BioRender (BioRender.com/s52a685). (**B**) Significant differences identified in various MEA metrics at three time points [14, 16 and 19 days *in vitro* (DIV14, DIV16 and DIV19)] between conditions: Genotype effect [significant difference between non-transgenic (NT) control knockdown and Huntington’s disease (HD) control knockdown cells], PIAS1 knockdown effect in HD cells (significant difference between control knockdown and PIAS1 knockdown in HD cells) and PIAS1 knockdown effect in NT cells (significant difference between control knockdown and PIAS1 knockdown in NT cells). Blue or yellow indicates significantly decreased or increased, respectively, values in the HD cells (compared to NT cells) and PIAS1 knockdown cells [compared to control siRNA (in either HD or NT cells)], respectively. ‘X’ indicates no significant difference detected. (**C**) Weighted mean firing rate (WMFR) was significantly higher in control knockdown-treated HD cells compared to control knockdown-treated NT cells at DIV14 and DIV16. This pattern reversed at DIV19. The increases seen in the WMFR in HD at DIV14 and DIV16 were significantly reduced upon knockdown with *Pias1* siRNA. Primary cortical neurons (PCNs) DIV14 WMFR: treatment, *F*(1,20) = 0.8446, *P* = 0.3690, genotype, *F*(1,20) = 12.11, *P =* 0.0024; PCN DIV 16 WMFR: treatment, *F*(1,20) = 13.63, *P* = 0.0014, genotype, *F*(1,20) = 14.75, *P* = 0.0010; PCN DIV 19 WMFR: treatment, *F*(1,20) = 1.243, *P* = 0.2781, genotype, *F*(1,20) = 2.793, *P* = 0.1102. *P < 0.05, ***P < 0.001. Graphs represent means ± standard error of the mean analysed by two-way ANOVA. A master analysis sheet of all significant differences in the remaining time points of PCNs can be found in the Supplementary material, File 016. *n* = 6 wells/condition.

MEA analyses of NT and HD R6/2 primary cortical neurons were carried out to test the impact of perturbing the SUMO network using PIAS1 knockdown. Cells were treated with either siRNA against *Pias1* or a scramble control siRNA, and functional measures of neuronal activity were recorded *in vitro* (see Fig. 6A for experimental workflow). Neuronal cultures for MEA were prepared from postsynaptic cortical components of the cortico-striatal circuit predominantly affected in HD. MEA analysis was performed on PCNs at various time points. The greatest numbers of changes in MEA metrics were observed at the time points DIV14, 16 and 19 and included alterations in the number of spikes, weighted mean firing rate (WMFR; Hz), inter-spike interval coefficient of variation (a measure of spike regularity), burst duration (s) and the number of spikes per burst (Fig. 6B and Supplementary material, File 016). Significant genotype effects were observed for all of the MEA measurements presented, except for number of bursts. The HD cells displayed significantly higher values at DIV14 and DIV16 compared to NT cells, suggesting hyperactivity at early time points. A reversal in directionality occurred in genotype effects by DIV19, where HD cells displayed significantly lower values than their NT counterparts. The change in the HD cells’ directionality supports the growing body of evidence indicating early hyperexcitability observed in HD.^89,90^ We also investigated whether perturbation by PIAS1 knockdown might impact electrophysiological neuronal activity. Since our previous work looking at PIAS1 knockdown in R6/2 mice revealed reversals in disease-related phenotypes in HD, we predicted that PIAS1 knockdown might decrease elevated neuronal activity in HD cells. PIAS1 knockdown rescued the hyperactivity in HD cells in several MEA measurements at DIV14 and DIV16, restoring those measurements to NT levels. No significant differences were observed with PIAS1 knockdown in HD cells at DIV19, but significantly lower values of the inter-spike interval coefficient of variation, burst duration and number of spikes per burst were seen in PIAS1 knockdown-treated NT cells. The changes we saw upon modulation of the SUMO-landscape with PIAS1 knockdown in neuronal cultures, using classic measurements by MEA analysis such as WMFR (Fig. 6C and Supplementary material, File 016), are consistent with our prior RNA sequencing data on the impact of PIAS1 knockdown in a full-length knock-in zQ175 mouse model^42^ and our analysis of the R6/2 SUMO proteome.

## Discussion

The deployment of discovery-based proteomic profiling of the SUMO proteome (SUMOylated protein and SUMO-associated proteins) continues to increase in the field.^89-91^ We evaluated changes to the brain SUMOome in neurodegenerative disease mice using an optimized technique sensitive enough to detect endogenous SUMO-enriched proteins. Importantly, our SUMO proteomic data indicated a strong relationship between SUMO and synaptic proteins, as well as dysregulation of SUMO networks in the transgenic expanded CAG repeat, truncated HTT HD model. The implications for this are broad, as dysregulation of synaptic activity is a hallmark of HD and other neurodegenerative diseases.

The changes in SUMO-enrichment of synaptic proteins fell into several functional themes, including synaptic vesicle docking, glutamatergic neurotransmission and MAP kinase signal transduction pathway-related proteins. For example, the SUMO proteomics dataset identified enrichment for a complex of proteins that interact with mGLUR7 (PICK1, guanine nucleotide-binding protein G subunits beta and gamma, and calmodulin).^92-94^ Notably, two events, mGLUR7 phosphorylation at serine residue 862 by protein kinase C (facilitates mGLUR7 SUMO conjugation) and mGLUR7 binding to PICK1, are critical for to stabilizing mGLUR7 expression^66,75^; and rodent studies suggest that the interaction of mGLUR7 and PICK1 is needed for proper neural function, as disruption of the mGLUR7a-PICK1 complex is sufficient to induce epilepsy-like seizures.^75,95^ PICK1 has not been shown previously in the literature as a target of SUMOylation, but our proteomics and *in vitro* SUMO assays confirmed its modification by SUMO. As such, we propose that the (de)SUMOylation of PICK1 may also be a requirement for PICK1 binding to mGLUR7, further enhancing or inhibiting the surface expression of mGLUR7, since SUMOylation is often used by the cell as a modification to direct protein subcellular relocalization.^96^ SUMOylation of PICK1 potentially highlights a mechanism through which SUMO may control the surface presence of receptors like mGLUR7; this pathway may be involved in the aberrant neuronal excitability and firing characteristic of HD pathology and will be studied further in future studies.

Proteins identified within the SUMO-enriched proteomics dataset have also been implicated in other areas of HD study. For instance, we recently showed that abnormal glucose and lipid metabolism is dysregulated in HD oligodendrocytes. PRKCE (protein kinase C epsilon), one of the nine proteins identified within the synaptogenesis signalling pathway and showing increased SUMO enrichment in our dataset, is a central gene dysregulated in HD mouse and human brain that appears to contribute to dysregulated oligodendrocyte maturation.^97^ Our proteomics dataset sampled whole striatal tissue, which on its own does not distinguish between different cell types of the brain; therefore, merging information from single-cell datasets and information across brain regions will contribute to our understanding of the cell type and regional specificity contributed by the SUMO network. To investigate a corresponding facet of synaptic dysfunction and activity in HD related to glutamatergic signalling and SUMO, we focused on mGLUR7 modification and localization. mGLUR7, like many glutamate receptors, can also serve as an autoreceptor to inhibit neurotransmitter release at the presynapse.^98-100^ Activation of group III receptors, such as mGLUR7, may potentially increase the viability of the GABAergic-projecting neurons in the striatum selectively lost in HD as a compensatory mechanism during disease progression. Alternatively, modulation of group III mGLUR activity may balance glutamatergic activity in the basal ganglia circuits during disease.^101^ Trafficking plays a critical role in modulating the spatiotemporal localization and activity of mGLURs.^102-107^ Excitotoxicity via glutamatergic signalling is a hallmark of HD, including signalling via metabotropic glutamate receptors.^108^ Enhanced levels of mGLUR7 surface expression may contribute to the glutamatergic signalling that occurs in HD cells and the downstream destruction resulting from glutamatergic excitotoxicity. Ligand-mediated activation can induce desensitization, promoting a negative feedback mechanism that protects receptors through internalization and preventing chronic receptor overstimulation.^109^ Reduced internalization of the mGLUR7 autoreceptor, inhibiting further glutamate release,^75,110^ may serve as a compensatory mechanism to reduce excessive neurotransmitter signalling.^106,107,111,112^ SUMOylation of mGLUR7 is a direct mechanism for regulating mGLUR7’s internalization or membrane expression, and here we show that the E3 SUMO ligase PIAS1 can facilitate its SUMOylation. Previous studies have shown that prevention of SUMOylation by a lysine-to-arginine mutation of mGLUR7 results in enhanced internalization of the mGLUR7 protein.^19^ We find in our targeted receptor internalization assays that knockdown of the PIAS1 protein in primary striatal neurons results in a similar effect. The results presented in this study indicate a potential mechanism by which mGLUR7 surface expression can be modulated through a post-translational modification. Depending upon the time point at which levels of mGLUR7 internalization are assessed, knockdown of a SUMOylating agent such as PIAS1 may prove to be beneficial for restoring the equilibrium of glutamatergic receptor localization and, subsequently, impact downstream glutamatergic signalling.

The glutamatergic signalling and neurotransmission proteins highlighted in numerous capacities within the SUMO proteomics dataset prompted our direct investigation of neuronal function in HD by MEA. We found significant genotype effects and PIAS1 knockdown rescued some hyperactivity in HD cells. One example protein that we hypothesize could be linked to the types of changes observed in the MEA analysis, mGLUR5, was also identified within four of the six pathways from the PINE visualization analysis and showed significantly increased enrichment in HD mouse striata. mGLUR5 plays a critical role in glutamatergic signalling, influencing synaptic function and modulating neuronal excitability. For example, pharmacological positive allosteric modulation of mGLUR5 by CDPPB in striatal slices rescues impaired long-term depression (LTD) in a mouse model of autism,^113^ and LTD formation is impaired in layer V of the medial prefrontal cortex upon mGLUR5 deletion.^114^ Interestingly, mGLUR5 and mHTT exhibit a functional interaction, and knockout of mGLUR5 induces hyperkinesia in mice, suggesting a potential role in HD chorea.^74,115^ SUMOylation of other glutamatergic proteins including mGLUR5 may also serve as a potential mechanism for widespread modulation of neuronal excitability; for instance, SUMOylation of the voltage-gated sodium channel (Na_v_1.2) enhances voltage-gated sodium currents in rat cerebellar granule neurons,^116^ whereas the SUMOylation of voltage-gated potassium channel (K_v_1.2) in hippocampal neurons regulates neuronal firing.^117^ Electrophysiological changes at the network level have also been documented in HD.^118^ Cortical pyramidal neurons of HD mice demonstrate significant hyperexcitability, which precedes the onset of behavioral symptoms.^83^ Although the later stages of disease characteristically display neuronal dysfunction and diminished activity,^119^ early stages and juvenile forms tend to show a hyperexcitable state,^120^ emphasizing the need for targeted reduction of neuronal activity in the early stages of disease.

Several proteins identified in the SUMO enrichment are supported by results from other groups using rodent brain tissue, HeLa cells and other systems.^70,71^ As expected, when the datasets are compared, the greater the similarities between method (enrichment of both covalent and non-covalent interactions) and source tissue (mouse), the greater the numbers of overlapping proteins identified between previous SUMO proteomic studies and the present study. These similarities, particularly in the enrichment of synaptic proteins and the dysregulation of this network in the R6/2 mouse brains, further highlight the importance of this network in regulating synaptic activity and the value of using this system to assess disease-related changes.

A limitation of our study is that an abundance of protein SUMOylation also occurs within the nucleus, regulating transcription, DNA damage repair and other processes, and our present study captured predominantly extranuclear/synaptic SUMO-associated proteins. Future work will aim to expand upon the results presented here and identify nuclear proteins more robustly, which may require purification of nuclei as a first step.

Collectively, the results demonstrate dysregulation of SUMO modification of synaptic proteins in HD and warrant further research into the specific mechanisms and consequences of protein SUMOylation in HD. We also identified SUMOylation of several other important classes of proteins, including mitochondrial, RNA-binding, and calcium-related proteins. Our findings provide evidence for the involvement of dysregulated SUMO networks in synaptic processes, potentially leading to enduring dysregulation of neurotransmission and neuronal activity in neurodegenerative disease. Finally, the targeted impact of PIAS1 knockdown on these measurements further supports the potential for modulation of PIAS1 and the SUMO network as a therapeutic target for the treatment of HD. Given the impact of SUMO modification broadly on neurodegenerative disorders, targeting the SUMO machinery may provide novel therapeutic approaches that can restore appropriate synaptic regulation.

## Supplementary Material

awae319_Supplementary_Data

## Data Availability

Proteomics data are deposited in the Proteomics IDEntifications Database (PRIDE): PXD046378 and the MASS spectrometry Interactive Virtual Environment (MassIVE): MSV000093185. All study data are included in the article and/or Supplementary material.
