# Supplementary material for: Dysregulation of protein SUMOylation networks in Huntington’s disease R6/2 mouse striatum
Source: Brain. 2024 Oct 11;148(4):1212–27. doi: 10.1093/brain/awae319 (PMC11969464; doi:10.1093/brain/awae319)
Supplement: awae319_Supplementary_Data [file awae319_supplementary_data.zip › brain-2024-01098-File008.pdf]

Supplemental Information

Supplemental Methods

Western blotting

Briefly, flash-frozen brain tissue was lysed and prepared for SUMO capture as described above. Whole cell lysates or SUMO-enriched elution were separated by sodium dodecyl sulfate-polyacrylamide gel electrophoresis (SDS-PAGE) using a Bolt 4-12%, 1.0 mm Bis-Tris gel (Invitrogen #NW04125BOX) paired with 20X MOPS running buffer (NuPAGE #NP0001). Proteins were transferred onto a 0.45 µM PVDF membrane (ThermoFisher Scientific #88518) or 0.45 µM nitrocellulose membrane (Bio-Rad #1620115). Near-infrared fluorescence of immuno-labeled proteins was assessed using an Odyssey Li-Cor DLx. SUMO-enriched protein output was normalised and tested for non-specific binding by measurement with a reversible total protein stain (Revert 700, Li-Cor Biosciences #926-11010) and compared to the amount of SUMO-enriched proteins identified by immunostaining with anti-SUMO1 and anti-SUMO2 antibodies. Protein quantification was calculated using Empiria Studio v2.0.

Plasmids

A pRK5-Myc-mGLUR7a plasmid clone was a gift from Katherine Roche, NIH/NINDS, Bethesda, MD, USA and YoungHo Suh, Seoul National University College of Medicine, Seoul, South Korea. The Myc-PICK1 cDNA clone was purchased from GenScript (OHu29966C) and subcloned into pCDNA 3.1(+)N-Myc. His-SUMO1 was a gift from Ron Hay, University of Dundee, Dundee, UK. Myc-PIAS1 cDNA clone in pCMV vector was purchased from SinoBiological (HG15957-NM).

Primary Neuronal Cultures

Six five-week-old female HD ovarian transplant females and six C57BL/6J/BCA male studs were purchased from Jackson Lab. Breeding pairs were combined for one week then separated. Postnatal day 0 (P0) pups were sedated on ice and decapitated to remove brains. Striatum and cortex were collected, and tails were genotyped. A protocol for culturing primary cortical and striatal neurons was adapted from the protocol as described in Vashishtha et al.[47] Briefly, primary cortical or striatal neurons were obtained from dissected tissues of six NT or six HD P0 pups. All procedures were performed under an IACUC-approved protocol. Dissected tissue was dissociated

with 2.5% trypsin and 50 mg/ml DNase I, triturated, centrifuged for 7 min at 300 *xg* at room temperature, and resuspended in 1-2 mL of culture medium designed for cells at days *in vitro* (DIV) 0-6 (Neurobasal A, 300 μM Glutamax, 1x antibiotic-antimycotic (AA), 25 μM β-mercaptoethanol, 1x B-27 supplement). Cells were counted and plated at a density of $1x10^6$ cells/mL in six-well plates coated with 100 mg/ml poly-D-lysine and 1 μg/ml laminin. On DIV2 cells were treated overnight with 1 μM arabinoside C to limit glial cell proliferation. Cells at DIV7 and older were cultured in maintenance media (BrainPhys Neuronal Medium [Stem Cell Technologies # 05790], 300 μM Glutamax, 1x AA, 1x B-27 supplement).

Receptor internalization assay

Primary striatal neurons at P0 were plated on glass coverslips in 24-well plates at $2.5x10^5$ cells/coverslip and treated with 7.5 μL of 100 μM Accell mouse *Pias1* siRNA SMARTpool (Horizon Discovery #E-059344-00-0020) in 750 μL 1x siRNA buffer, or the Accell non-targeting control pool (Horizon Discovery #D-001910-10-20), at DIV5. Between DIV11-12 cells were transfected with c-Myc-tagged mGLUR7 (Myc-mGLUR7) using an adapted method of calcium phosphate co-precipitation[49] and incubated for 24 hrs. In order to differentiate between surface-expressed and internalised Myc-tagged mGLUR7, a receptor internalization assay was adapted from Choi et al.[19] Neurons were washed with PBS and incubated with 2 μg/ml c-Myc primary antibody (Millipore Sigma #05-419) for 10 min at room temperature to label Myc-tagged mGLUR7. Cells were washed with PBS and returned to the conditioned media at 37°C for 15 min and washed again with PBS. Neurons were fixed with 4% paraformaldehyde/4% sucrose in PBS for 20 minutes, washed with PBS, and blocked with 10% normal goat serum for 1 hr. Surface-expressed receptors were labeled by staining with Alexa Fluor 568 goat anti-mouse IgG (1:500 # A11004) for 30 minutes at room temperature (color signals were converted to magenta. Neurons were washed with PBS and permeabilised with 0.25% Triton-X-100 for 5 minutes, washed with PBS, and blocked with 10% normal goat serum for 1 h. Internalised receptors were then labeled by staining with Alexa Fluor 488 goat anti-mouse IgG secondary antibody (1:500#A11029) for 30 minutes at room temperature (color signals were converted to green). Neurons were then washed a final time with PBS and mounted with ProLong Antifade Kit (Invitrogen # P36930). Maximum projection images were obtained at 63x 1.3 using a Zeiss LSM 900 Airyscan 2 confocal microscope.
