# Supplementary material for: Dysregulation of protein SUMOylation networks in Huntington’s disease R6/2 mouse striatum
Source: Brain. 2024 Oct 11;148(4):1212–27. doi: 10.1093/brain/awae319 (PMC11969464; doi:10.1093/brain/awae319)
Supplement: awae319_Supplementary_Data [file awae319_supplementary_data.zip › brain-2024-01098-File009.pdf]

Supplementary figures

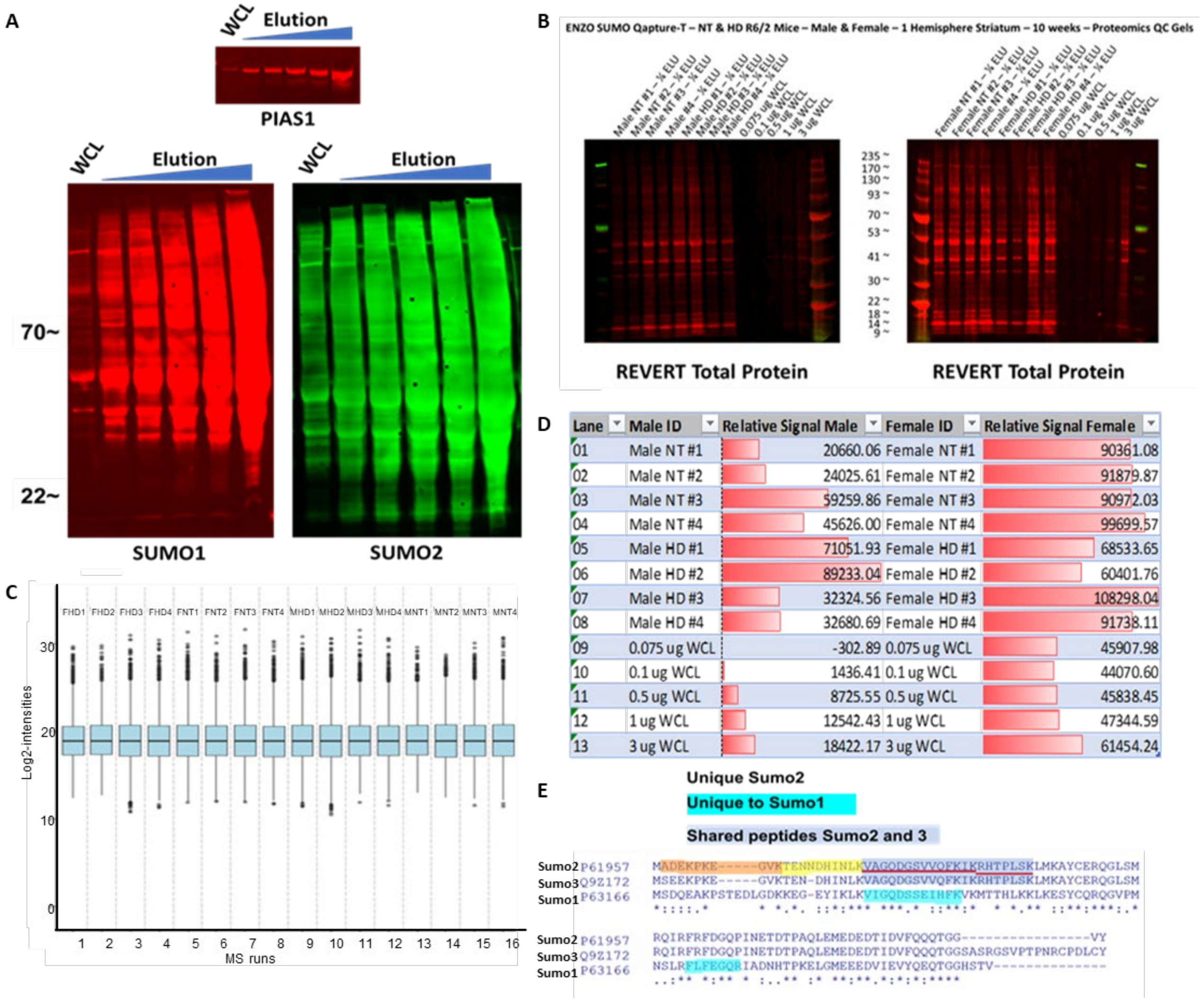

Supplementary Figure 1 – Detection of differentially SUMO-enriched protein from R6/2 HD mouse brain by mass spectrometry. (A) Increasing detection of a SUMOylated protein, PIAS1, demonstrates proportional increase in SUMOylation of a target protein (top). Increasing concentration of input material represents proportional increase in total SUMOylated protein capture for both SUMO1 and SUMO2 immuno-stained western blots (bottom left and bottom right, respectively). (B) Total protein stain and quality control gel showcasing relative amount of SUMO elution:trypsin ratio used for mass spectrometry-based analysis. (C) Intensity following normalization demonstrated a median distribution across samples. (D) Quantification by Li-Cor images of quality control gels for SUMOylated protein input for proteomic analysis. (E) Detection of SUMO1 and SUMO2 peptides in proteomic analysis of striatal mouse tissue. Identified spectra

indicate the successful detection of SUMO peptide spectrum matches. Asterisks denote shared amino acids across SUMO1, 2, 3 and dots represent number of shared amino acids. WCL: whole cell lysate

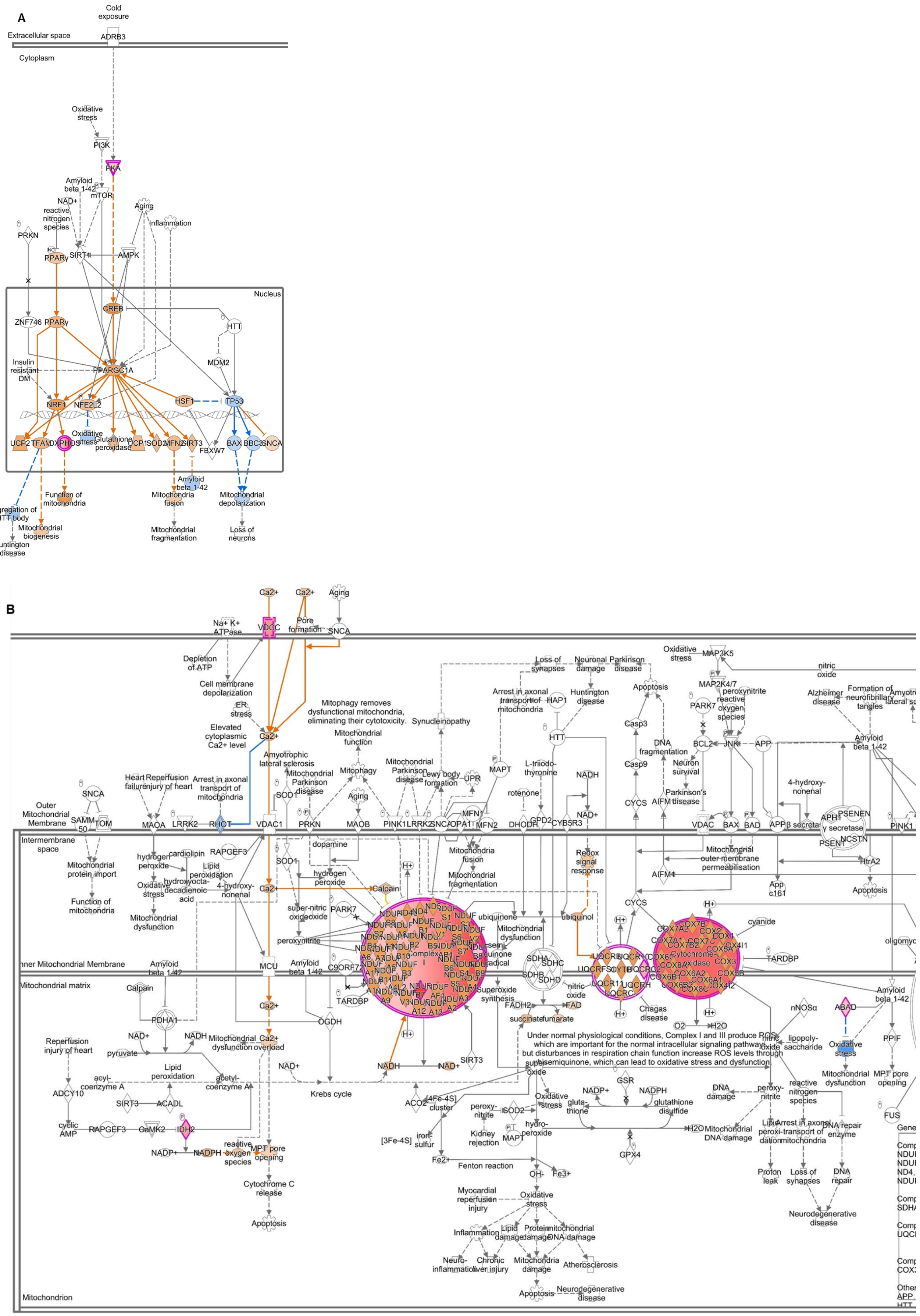
A
Cold exposure
Extracellular space
ADRB3
Cytoplasm
Oxidative stress
PI3K
PKA
Amyloid beta 1-42
mTOR
NAD+
reactive nitrogen species
Aging
Inflammation
PRKN
PPARγ
SIRT1
AMPK
Nucleus
CREB
ZNF746
HTT
MDM2
Insulin resistant DM
PPARGC1A
NRF1
NFE2L2
HSF1
TP53
UCP2
TFAM
OXPHOS
Oxidative stress
Glutathione peroxidase
UCP1
SOD2
MFN2
SIRT3
FBXW7
BAX
BBC3
SNCA
Amyloid beta 1-42
Aggregation of HTT body
Function of mitochondria
Mitochondria fusion
Mitochondrial depolarization
Mitochondrial biogenesis
Huntington disease
Mitochondrial fragmentation
Loss of neurons
B
Ca2+
Aging
Na+ K+ ATPase
VDCC
Pore formation
SNCA
Depletion of ATP
Cell membrane depolarization
ER stress
Elevated cytoplasmic Ca2+ level
Mitophagy removes dysfunctional mitochondria, eliminating their cytotoxicity.
Mitochondrial function
Synucleinopathy
Loss of synapses
Neuronal damage
Parkinson disease
Arrest in axonal transport of mitochondria
HAP1
Huntington disease
Apoptosis
Oxidative stress
MAP3K5
MAP2K4/7
peroxynitrite
reactive oxygen species
nitric oxide
Alzheimer disease
Formation of neurofibrillary tangles
Amyloid beta 1-42
Amyotrophic lateral sclerosis
Mitochondrial Parkinson disease
Mitophagy
Heart failure
Reperfusion injury of heart
Lewy body formation
UPR
MAPT
L-triiodothyronine
NADH
HTT
Casp3
DNA fragmentation
PARK7
BCL2
JNK
APP
Casp9
Neuron survival
4-hydroxy-nonenal
Outer Mitochondrial Membrane
SAMM50
TOM
MAOA
LRRK2
RHOT
VDAC1
PRKN
MAOB
PINK1
MFN1
MFN2
OPA1
rotenone
DHODH
GPD2
CYB5R3
NAD+
CYCS
AIFM
Parkinson's disease
VDAC
BAX
BAD
APP
β secretase
γ secretase
APH1
PSENEN
PSEN1
NCSTN
Intermembrane space
hydrogen peroxide
cardiolipin
RAPGEF3
Lipid peroxidation
hydroxyocta-decadienoic acid
4-hydroxy-nonenal
dopamine
SOD1
Mitochondrial protein import
Function of mitochondria
Mitochondrial dysfunction
Calpain
super-nitric oxideoxide
PARK7
Mitochondria fusion
Mitochondrial fragmentation
Redox signal response
Mitochondrial outer membrane permeabilisation
AIFM1
HtrA2
App c161
ubiquinone
ubiquinol
cyanide
Inner Mitochondrial Membrane
Amyloid beta 1-42
MCU
C9ORF72
TARDBP
SDHA
SDHC
SDHB
SDHD
semiquinone radical
Superoxide synthesis
nitric oxide
UQCRH
COX7A2
COX7B
COX2
COX1
Cytochrome-c oxidase
COX3
TARDBP
Mitochondrial matrix
Calpain
PDHA1
OGDH
FADH2
FAD
succinate
fumarate
Chagas disease
O2
H2O
nNOSα
ABAD
Amyloid beta 1-42
PPIF
Reperfusion injury of heart
NAD+
NADH
pyruvate
Mitochondrial dysfunction
Ca2+ overload
Lipid peroxidation
acyl-coenzyme A
acetyl-coenzyme A
Krebs cycle
SIRT3
Under normal physiological conditions, Complex I and III produce ROS which are important for the normal intracellular signaling pathways but disturbances in respiration chain function increase ROS levels through superoxide semiquinone, which can lead to oxidative stress and dysfunction.
lipopoly-saccharide
Oxidative stress
Mitochondrial dysfunction
MPT pore opening
ADCY10
SIRT3
ACADL
ACO2
[4Fe-4S] cluster
GSR
NADPH
NADP+
glutathione disulfide
glutathione
peroxynitrite
SOD2
DNA damage
reactive nitrogen species
cyclic AMP
RAPGEF3
CaMK2
IDH2
NADP+
NADPH
reactive oxygen species
MPT pore opening
[3Fe-4S]
iron-sulfur
Fe2+
Kidney rejection
MAPT
hydrogen peroxide
H2O
GPX4
Mitochondrial DNA damage
Lipid peroxidation
Arrest in axonal transport of mitochondria
DNA repair enzyme
FUS
Fenton reaction
Fe3+
Cytochrome C release
OH-
Proton leak
Loss of synapses
DNA repair
Apoptosis
Myocardial reperfusion injury
Oxidative stress
Neurodegenerative disease
Inflammation
Lipid damage
Protein damage
mitochondrial DNA damage
Neuro-inflammation
Chronic liver injury
Mitochondria damage
Atherosclerosis
Apoptosis
Neurodegenerative disease
Mitochondrion

Supplementary Figure 2: Ingenuity Pathway Analysis of the HD vs NT SUMO DEPs Mitochondrial dysfunction canonical pathway. Mitochondrial dysfunction was one of the canonical pathways that was significant in the analysis of the DEPs it was predicted to be inhibited with a negative z score A) Nuclear localised genes related to mitochondrial dysfunction B) Mitochondrial localised genes related to mitochondrial dysfunction Darker orange colors indicate greater predicted activation, while darker blue colors indicate greater predicted inhibition.

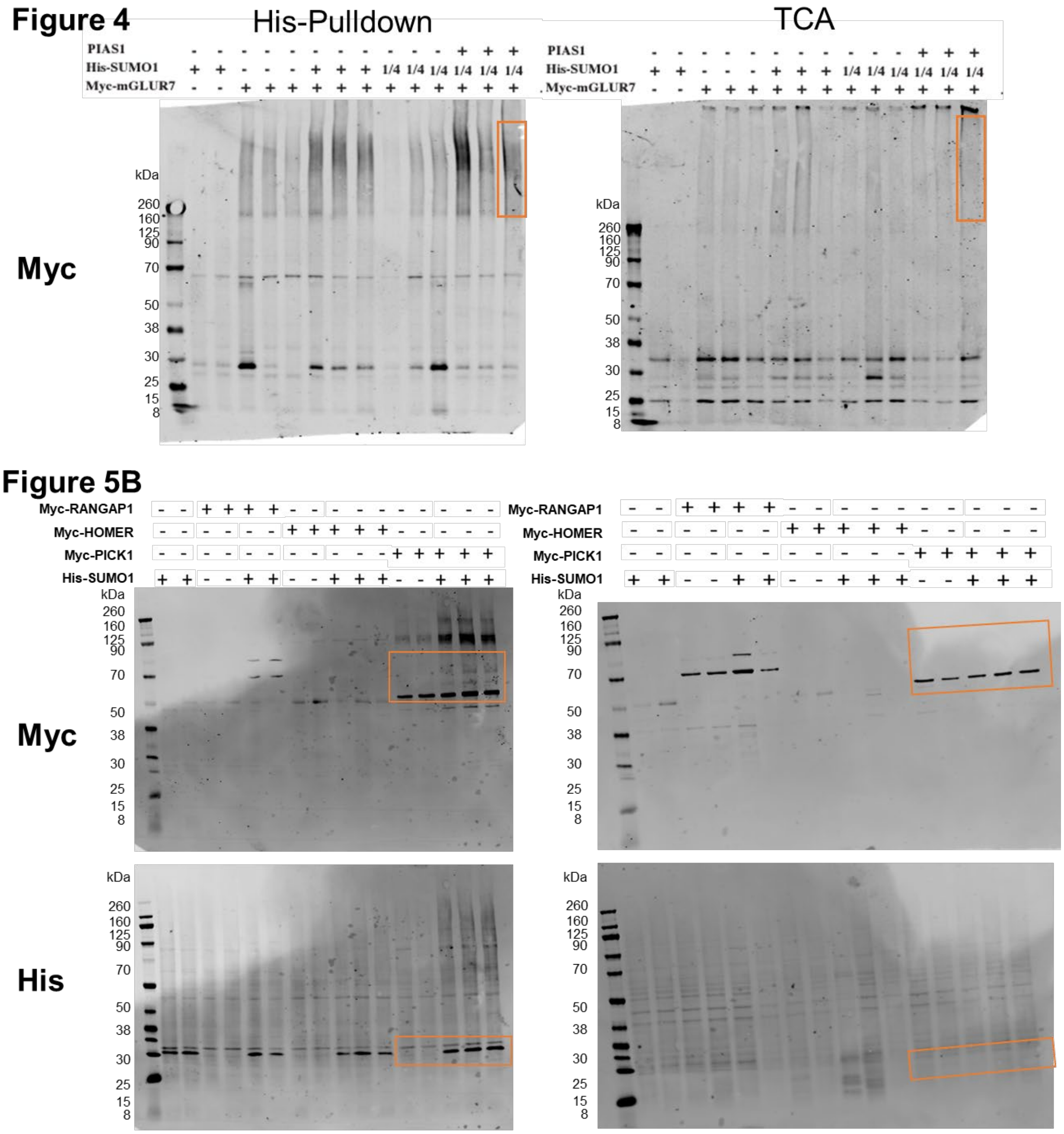

Supplementary Figure 3: Whole western blots for what is shown in **Fig 4** probing for the Myc-tagged proteins and **Fig 5B** probing for Myc-tagged proteins and His-SUMO1 modified proteins

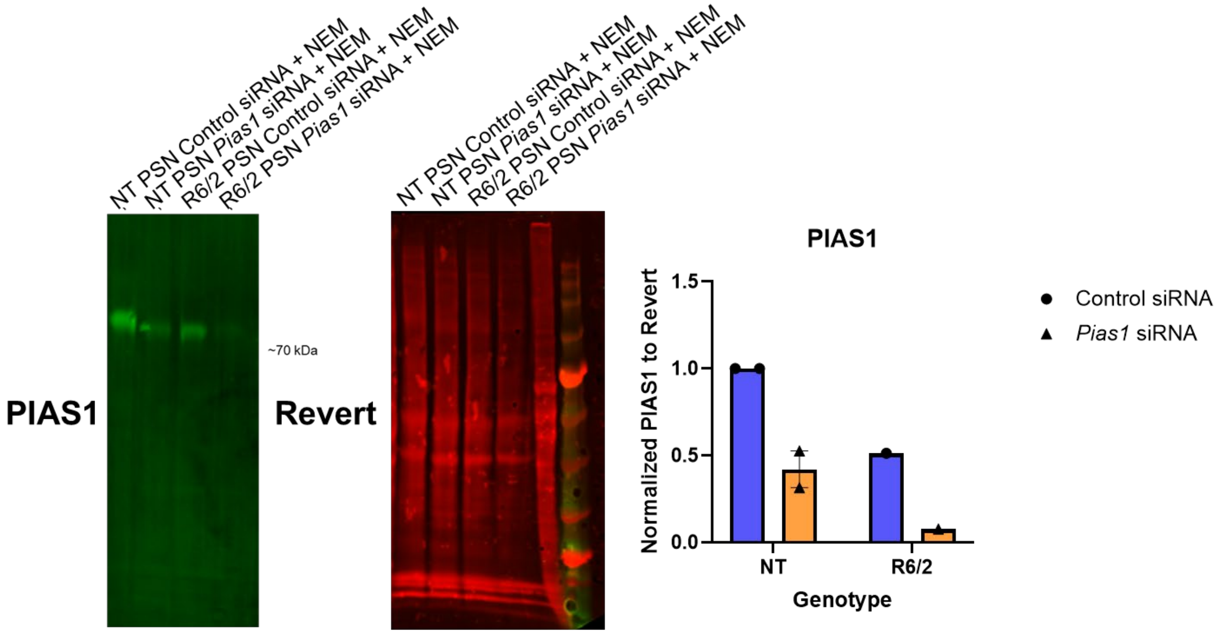

Supplementary Figure 4: Primary striatal neurons (PSN) were harvested and pelleted to assess levels of successful siRNA-mediated *Pias1* KD within mGLUR7 receptor internalization assay experiments. Successful KD of PIAS1 protein was quantitated by western blot and calculated to be ~68% within NT cells, and ~84% within R6/2 cells. NEM: N-ethyl-maleimide.

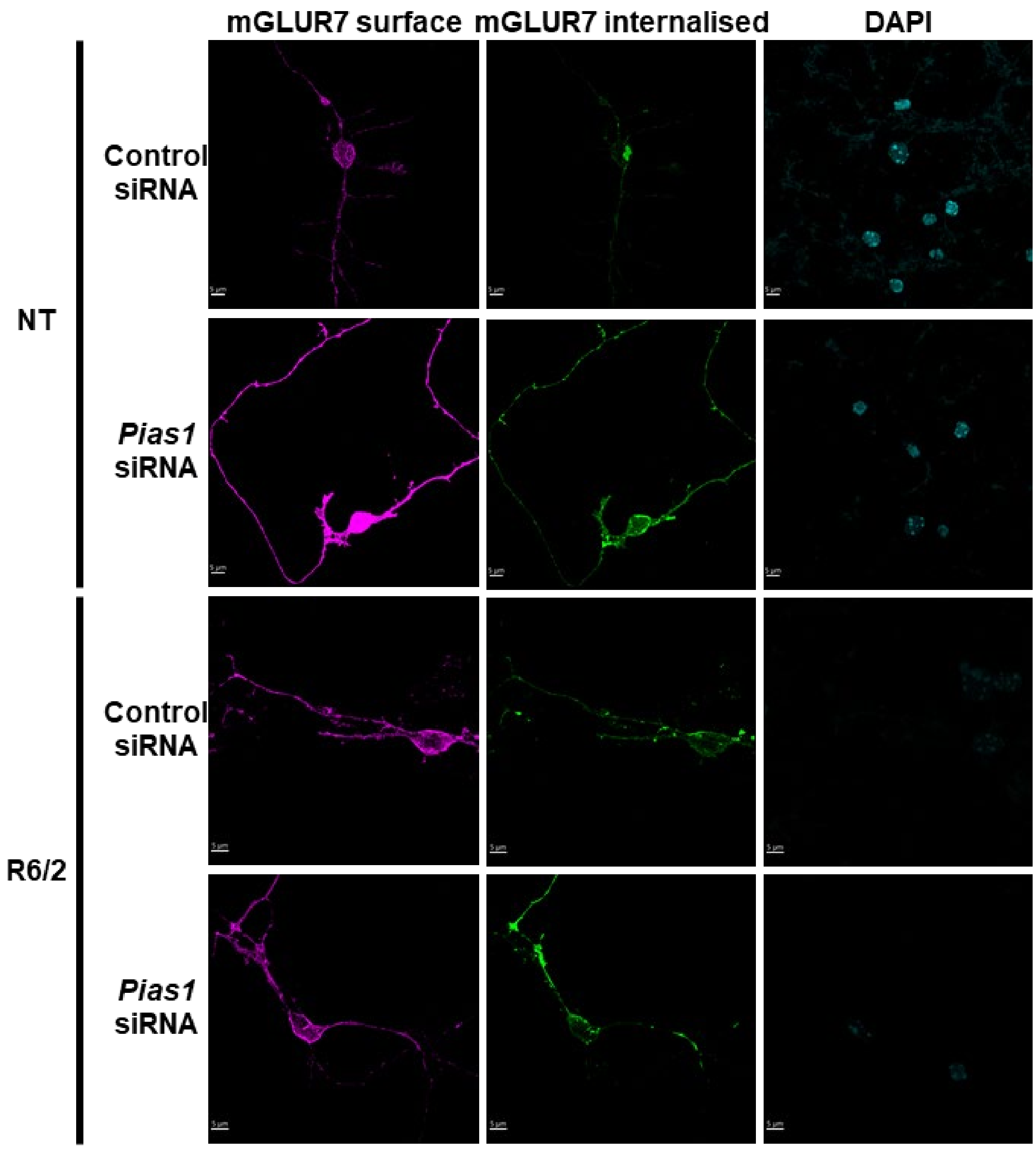

Supplementary Figure 5: Immunofluorescence of primary striatal neurons for the mGLUR7 receptor showing internalised (green) and external surface (magenta) expression along with nuclei staining with DAPI, images are individual channels from **Fig 5D**. Images were taken on Zeiss LSM 900 Airyscan 2 confocal microscope

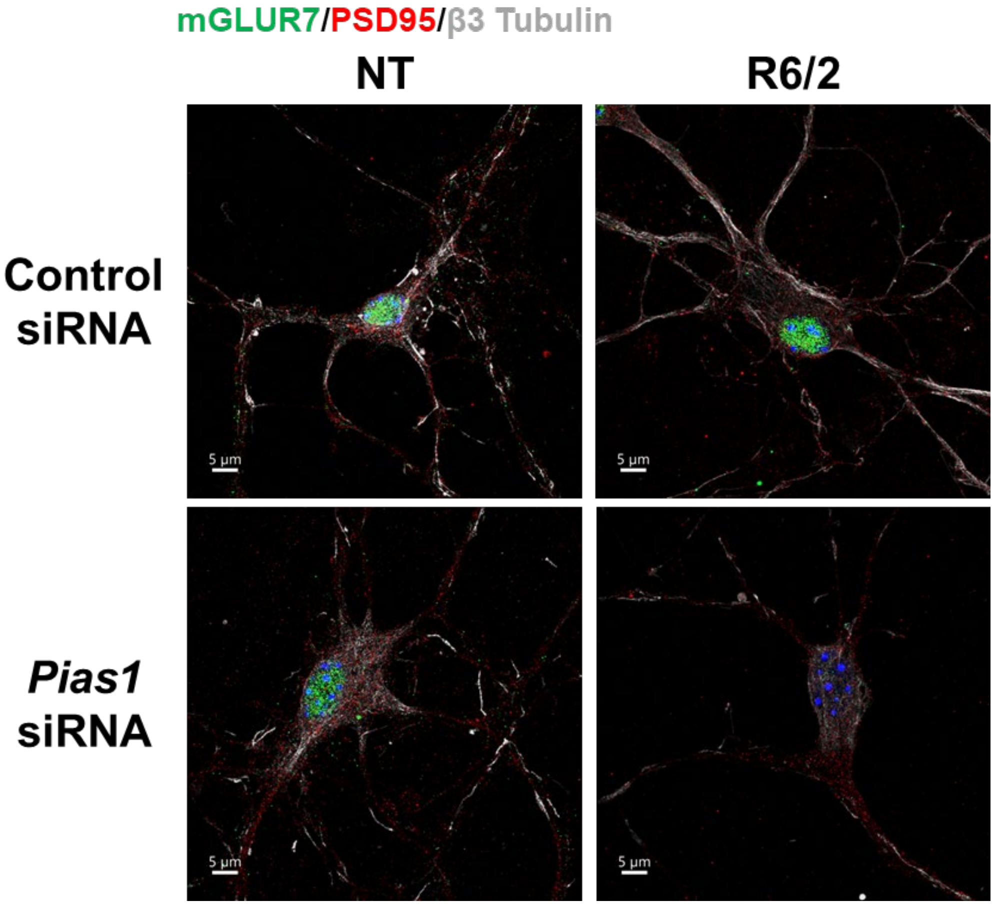

Supplementary Figure 6: Immunofluorescence staining of the primary cortical neurons. mGLUR7 in green, PSD95 in red, nuclei in blue and βIII tubulin in white. Images were taken on Zeiss LSM 900 Airyscan 2 confocal microscope
